# The impact of paid family leave on parent and infant health outcomes: a systematic review of quasi-experimental research

**DOI:** 10.1186/s12889-026-27709-2

**Published:** 2026-07-14

**Authors:** Laura Hergenrother, Sara Honaker, Meghan Shanahan

**Affiliations:** 1 Department of Maternal and Child Health, University of North Carolina at Chapel Hill, 135 Dauer Drive, 170 Rosenau Hall, CB #7400, Chapel Hill, NC 27599-7400 United States; 2 Department of Maternal and Child Health, University of North Carolina at Chapel Hill, 137 E. Franklin Street, Ste. 500, Rm. 500-F, Chapel Hill, NC 27599-7505 United States

**Keywords:** Paid Family Leave (PFL), Parent Health Outcomes, Infant Health Outcomes, Health Inequities, Maternal and Child Health

## Abstract

**Background:**

Despite strong international evidence that Paid Family Leave (PFL) policies support the physical health and mental health of parents and infants and domestic evidence of its benefits on family economic well-being, the United States lacks a national PFL policy.

**Methods:**

This systematic review sought to evaluate the association of PFL in the United States with parent and infant health outcomes. Quasi-experimental research studies with comparisons of states with PFL policies versus those without such policies were eligible for review. A search strategy was created to match the research aims, and PubMed, CINAHL, Scopus, and PsycINFO were searched. Included studies were assessed by a title and abstract screening, followed by a full-text screening completed by two reviewers. Data were abstracted and narratively synthesized.

**Results:**

All included studies found improvements in birthing people’s health outcomes for those exposed to PFL compared to those not exposed. All studies found positive or neutral benefits of PFL for non-birthing partners’ and infants’ health outcomes. Health outcomes were measured as physical, mental, and well-being outcomes for parents, and physical health outcomes for infants. PFL was associated with a positive effect on birthing people’s physical health via: self-reported health status, number of nights spent in the hospital, the likelihood of reporting sick days, and daily prescription drug use; mental health via postpartum psychological distress; and well-being via: the likelihood of being overweight and alcohol consumption. Findings were less pronounced for non-birthing partners. PFL was associated with improvements in infant health via: the post-neonatal mortality rate, parent-reported overall infant health, abusive head trauma admissions, and hospital admissions for upper respiratory and gastrointestinal infections.

**Conclusions:**

Although PFL was found to be associated with a variety of health impacts, diverse groups of parents and infants experienced different magnitudes of treatment effects. Future work is needed to better understand the extent to which PFL is associated with health inequities at the population level. Analyses are recommended to span across social identities.

**Supplementary Information:**

The online version contains supplementary material available at https://doi.org/10.1186/s12889-026-27709-2.

## Background

Despite compelling evidence demonstrating the beneficial impact of Paid Family Leave (PFL) on parents and infants, the United States (U.S.) lacks a national PFL policy. As of 2026, only nine states (CA, CO, CT, MA, NJ, NY, OR, RI, WA) and D.C. have implemented mandatory PFL policies, with policies scheduled to be implemented in four other states (DE, MD, ME, MI) between 2026 and 2028 [1, 2]. State-sponsored PFL policies are distinct from the national Family and Medical Leave Act (FMLA), which provides unpaid, job-protected leave for qualified employees. These policies also differ from employer-provided paid leave benefits, which vary across all states, including states without PFL policies. There is considerable variation in the design of state-sponsored PFL policies including different wage replacement levels, length of leave, job protection and other policy characteristics (Additional File 1).

Across the U.S., PFL is more accessible to white and Asian birthing people than it is for those who are Black and Hispanic [3]. Specifically, Asian, Hispanic, and Black birthing people respectively received 0.9, 2.0, and 3.6 fewer weeks of full-pay leave than white birthing people [3]. Access to PFL varies across demographics outside of race and ethnicity, with a higher percentage of those with college degrees, higher incomes, and those who work full-time receiving this benefit [4]. Not only does PFL access vary across social identity, so does PFL use, particularly among racially minoritized and low-income groups [3]. It is important to assess the impacts of PFL within the U.S. policy context, where fragmented unpaid and paid leave policies vary across states, and PFL is the primary mechanism for some groups to gain access to paid time away from employment.

Internationally focused research provides robust evidence of PFL’s positive health benefits. Findings from 35 Organisation for Economic Co-operation and Development countries suggest that longer PFL durations are associated with improved neonatal mortality, infant mortality, and under-five mortality rates by 1.9 to 5.2% [5]. Research in Norway suggests improvements in birthing people’s blood pressure, pain, and mental health status in addition to increases in health-promoting behaviors such as exercise and smoking cessation associated with PFL [6]. Furthermore, PFL policies in Europe have been associated with a 0.38-point reduction in the Euro-D scale for late-life depressive symptoms for birthing people [5].

Much of the existing research base in the U.S. focuses on PFL’s positive socioeconomic impacts, such as increases in employment and wages [7–10]. A small but growing body of literature focuses on PFL’s health impacts for parents and infants. Several studies consider the mechanisms through which PFL impacts parent and infant health; compared to unpaid leave or no leave, PFL is linked to decreases in parents’ stress levels during pregnancy and the postpartum period through increased financial security, job security and food security, which lead to positive impacts on parent and infant health [8, 10–12]. Previous reviews on PFL’s health impacts consider different research designs and draw from vast bodies of literature [13–15]. One systematic review investigated the association between PFL and birthing people’s health, at once finding PFL to be beneficial to parent mental health and noting limited comparability across studies due to differences in study design, definitions of exposure and outcome variables [13]. Another found a variety of benefits attributed to PFL, including improvements in postpartum depression, intimate partner violence, infant attachment, infant mortality, birthing person and infant rehospitalizations, pediatric visit attendance, timely administration of infant immunizations, and breastfeeding initiation and duration [15]. Evidence based in the U.S. also focuses on the duration of leave; for example, the association between leave duration and depressive symptoms has been described as “U-shaped,” with a minimum at six months, suggesting that improvements in mental health are greatest when birthing people take six months of paid leave [16]. Finally, many existing systematic reviews focus primarily on mental health outcomes for birthing people [13, 17].

### Aims and objectives

The need for the present systematic review arose from the absence of a recent investigation into PFL’s association with both parent and infant health outcomes in the U.S with comparability across research design and a focus beyond the duration of leave. To this end, we did not examine leave duration but instead assessed how the presence versus absence of state-sponsored PFL policies affects health outcomes, allowing for a broader and more holistic understanding of PFL’s impacts. Additionally, to our knowledge, no systematic reviews on PFL’s association with both parent and infant health outcomes in the U.S. have been recently published.

This review aimed to address how the presence of state-sponsored PFL policies in the U.S. is associated with the health outcomes of birthing people, non-birthing partners, and infants under the age of one year in states with PFL policies compared to that of states without state-sponsored PFL policies.

## Methods

### Data source and search strategy

We conducted a systematic literature review using an electronic database search of PubMed, CINAHL, Scopus, and PsycINFO in March 2024. Under the guidance of a University of North Carolina at Chapel Hill Health Sciences librarian, we applied a standardized combination of Boolean operators with keywords capturing the different search elements. Studies were searched using free-text keyword searches of titles and abstracts instead of Medical Subject Headings (MeSH) terms to ensure inclusion of recently published studies that may not have been indexed with MeSH terms.

The subsequent search strategy was designed to include all published articles which considered the relationship between the following elements: (1) family leave policies (2), policies that are paid (3), birthing people, non-birthing partners, and infants under the age of one year. Specific outcomes, setting and time were excluded from the search strategy to avoid overly narrowing the articles captured in the search. The following search terms for family leave were used: “paid leave,” “parental leave,” “family leave,” “maternity leave,” and “paternity leave,” for paid: “paid” “job protected,” “employer paid,” “state sponsored,” and for our population of interest: “infant,” “family,” and “parent.” A full list of search terms can be found in Additional File 2. We modified search term formats to be compatible with each database using the Polyglot tool. Searches were merged and de-duplicated via Covidence with manual checking.

This study did not require IRB approval as it did not involve human subjects and was based entirely on publicly available literature. This review was conducted and reported in accordance with the PRISMA 2020 guidelines, and the completed checklist is provided in Additional File 3.

### Eligibility criteria

We evaluated all studies against this review’s SPICE (Setting, Perspective, Intervention, Comparison, Evaluation) criteria [18]:

- Setting: the U.S. since 2004
- Perspective: Birthing people, non-birthing partners, and infants under the age of one year
- Intervention: State-sponsored PFL policies
- Comparison: States with PFL policies vs. states without these policies
- Evaluation: Parent and infant health outcomes

Studies were eligible for inclusion if they met the inclusion and exclusion criteria outlined in Table 1. Studies were also included only if they were published after 2004, the year that the first state (CA) enacted its PFL policy. Studies were included if they assessed the implementation of state-sponsored PFL policies, without attention to the level of wage replacement, length of leave, or any other policy characteristic. States with PFL policies includes states with active, mandatory PFL polices (listed in Additional File 1). The absence of state-sponsored PFL policies includes states with unpaid leave such as FMLA, employer-provided leave benefits, voluntary PFL programs, and enacted but inactive mandatory PFL policies. Health outcomes were included if they related to the physical and mental well-being of parents and the physical health of infants; studies that investigated socioeconomic outcomes, gender equality, or rates of access to PFL were not included.

**Table 1:** Inclusion and Exclusion Criteria

Inclusion Criteria	Exclusion Criteria
• Published in English • Based in the United States • Peer-reviewed • Uses a quasi-experimental design • The study population includes parents and infants • Examines state-sponsored PFL policies • Evaluates health outcomes	• Not published in English • Not based in the United States • Not peer-reviewed (e.g., commentaries, dissertations, editorials, perspectives, conference proceedings, working papers etc.) • Does not use a quasi-experimental design (e.g. observational studies, descriptive studies, case studies, etc.) • The study population does not include parents or infants (e.g. children, non-parental caregivers, families only, etc.) • Does not examine PFL (i.e., examines unpaid leave, sick leave, safe leave, annual leave, bereavement leave, etc.) • Does not evaluate health outcomes (i.e., socioeconomic outcomes, gender equality and rates of PFL access)

### Study screening

Study screening followed PRISMA guidelines. Included studies were first assessed independently by title and abstract screenings conducted by two reviewers (LH and SH). Each study was assessed against the eligibility criteria during this screening. The reviewers agreed on the majority of studies during the title and abstract screening; discrepancies were resolved through discussion prior to full text review. Full text reviews were conducted by the same two reviewers. After this step, we hand searched references from the included studies to identify potentially relevant studies that were not captured by the database search.

### Data abstraction

All studies that met full eligibility criteria and passed the full text screening were assessed with a data abstraction form found in Additional File 4 (LH). This form was designed to align with this systematic review’s aims. Abstraction domains included citation details; study characteristics such as aims, design, population, data source(s), and data analysis method(s); state(s) of interest; measures of health outcomes; main findings, conclusions, implications for practice, policy, and future research; and study limitations.

### Data synthesis

Only aggregate (study-level) data from included studies was used. Relevant study details, including sample size, state of interest, time period and data source, intervention and control, health outcomes measured, and main findings, were extracted and compiled in tables during data abstraction; these tables guided data synthesis, and a narrative description was produced.

### Risk of bias

Risk of bias was assessed using version 2 of the ROBINS-I (Risk-Of-Bias In Non-randomized Studies – of Interventions) tool. This tool evaluates non-randomized studies across six domains including: bias due to confounding; bias due to the classification of the intervention; bias due to the selection of participants; bias due to missing outcome data; bias due to the measurement of the outcome, and bias due to the selection of the reported result. Overall risk of bias is categorized as low, moderate, serious, or critical. All included studies were assessed using this framework.

## Results

### Study screening

From the initial database search, 5,543 studies were captured and 2,456 were removed as duplicates, leaving 3,087 studies for title and abstract screening. From this screening, 3,056 were removed as their title and abstracts did not meet eligibility criteria, leaving 31 studies for full text review. Both reviewers agreed that 21 articles under full text review did not meet full eligibility criteria and ten did. This information can be found in Additional File 5.

### Study characteristics

Study characteristics are summarized in Table 2. Ten studies met full eligibility criteria [19–28]. All studies were published between 2016 and 2024, and all data used were collected between 1993 and 2019. Eight studies investigated the impacts of California’s Paid Family Leave (CA-PFL) policy, one looked at NY’s PFL policy [27] and another considered the impacts of both CA and NJ’s PFL policies [22].

**Table 2:** Data Summary Table

Study	Sample Size	States of Interest	Time Period and Data Source	Intervention and Control	Health Outcome Measured	Main Findings
1. Bullinger (2019) [19]	*n* = 22,050	California	2003, 2007, 2011–2012 / *National Survey of Children’s Health (NSCH)* and 2001–2007 / *Behavioral Risk Factor Surveillance System (BRFSS)*	CA-PFL vs. non-PFL neighboring states (Control 1 = NV, AZ, WA), non-PFL big states (Control 2 = TX, FL, NY, PA), all non-CA states plus D.C. (Control 3)	Parent-reported overall infant health, infant food allergy, infant respiratory allergy, and parent mental health	o Access to CA-PFL improved overall child health by 9% points (pp) (0.086, *p* = 0.000) relative to neighboring states, 5 pp (0.048, *p* = 0.169) relative to other large states, and 7 pp (0.074, *p* = 0.000) relative to all non-PFL states. o CA-PFL was suggested to improve birthing person mental health status by up to 18 pp (0.177,*p* = 0.002) relative to other large states. o Non-birthing partners were not found to experience any improvements in mental health status due to PFL. o Due to CA-PFL, low-income households were more likely to report improvements in maternal mental health (*p* = 0.000). o Infants showed a reduction in asthma by 2.7 pp (-0.027, *p* = 0.013) relative to neighboring states and all non-PFL states. o There were no significant effects of PFL on respiratory allergies or food allergies. o CA-PFL had no effect on low-birthweight, preterm birth, or gestation length.
2. Chen et al. (2023) [20]	*n* = 5,508	California	2000–2008 / *National Vital Statistics Survey (NVSS)*	CA-PFL vs. all non-CA states plus D.C.	Post-neonatal mortality rate [PNMR] (defined as infant deaths between 28 and 365 days per 1000 live births)	o PFL reduced PNMR by 0.135 (*p* = 0.000), or approximately 339 infant lives. o There were fewer deaths from health-related causes and larger effects for infants with married mothers and for infant boys.
3. Doran et al. (2020) [21]	*n* = 7,379	California	2000–2010 / *National Health Interview Survey (NHIS)*	CA-PFL vs. “Synthetic California” (Abadie et al., 2010; Abadie; Cattaneo, 2018).	Postpartum psychological distress (PPD) for birthing people	o Access to PFL was associated with a 0.636-point decrease point (95% CI = − 1.202, − 0.070) in PPD symptoms among birthing people with infants. o CA-PFL was found to hold more benefits for Black and Hispanic birthing people (compared to white birthing people) and non-college-educated birthing people (compared to college-educated birthing people) o Birthing people who were young, single, and U.S.-born, were shown to benefit more from CA-PFL than their counterparts.
4. Irish et al. (2021) [22]	*n* = 28,638	California and New Jersey	1997 − 2016 / *National Health Interview Survey (NHIS)*	PFL in CA and NJ vs. all non-PFL states (except RI)	Changes in parent psychological distress	o Exposure to PFL was associated with a 0.49-point decrease in psychological distress among parents (95% CI = − 0.770, − 0.21). o In stratified models, white, middle income, younger, and unmarried parents experienced improvements in psychological distress. o Black and Hispanic parents experienced increased psychological distress relative to white parents o Middle-income parents experienced greater improvements in psychological distress relative to low-income parents
5. Kim (2024) [11, 23]	N/A	California	1996–2013 / *Survey of Income and Program* *Participation (SIPP)*	CA-PFL vs. other large states (Control 1 = FL, NY, PA, TX), Control 1 + neighboring states (Control 2 = Control 1 + AZ, NV, OR, WA), all non-CA states plus D.C. (Control 3)	Self-reported health status, number of nights spent in the hospital, any sick days, and daily prescription drug usage for parents	o Birthing people with access to CA-PFL showed an increase in self-reported health being “excellent” by 24 pp (0.243, *p* = 0.000). o Birthing people also showed a reduced likelihood of reporting any days sick (-6.51, *p* = 0.000), fewer nights in the hospital (-0.058, *p* = 0.000) and a lower likelihood of prescription drug use (-0.389, *p* = 0.000). o Birthing people with access to CA-PFL spend 0.6 fewer nights in the hospital compared to those not exposed to PFL. o For non-birthing partners, CA-PFL led to an increase in “excellent” health by eight to 15 pp (0.149, *p* = 0.000). o Non-birthing partners showed a reduced likelihood of reporting any sick days (-0.128, *p* = 0.000) but no change in the number of days spent in the hospital (0.455, *p* = 0.064) or the rate of prescription drug use (0.008, *p* = 0.699).
6. Klevens et al. (2017) [24]	N/A	California	1995–2011 / *Statewide Inpatient Databases (SID) from the Healthcare Cost and Utilization Project*	CA-PFL vs. seven non-PFL states (AZ, CO, FL, IA, MD, MA, WI)	Abusive Head Trauma (AHT) admissions among infants < 1 year old per 100,000	o CA-PFL policy was significantly associated with a level decrease of 5.11 (*p* = 0.035) in the AHT admissions per 100,000 infant < 1 year old. o While comparison states experienced increases in AHT rates during the years known as the Great Recession, CA did not.
7. Lee et al. (2020) [25]	*n* = 6,690	California	1993–2017 / *Panel Study of Income Dynamics (PSID)*	CA-PFL vs. non-PFL states	Respondent-rated health, psychological distress, overweight and obesity, and alcohol use for parents	o Birthing people and non-birthing partners saw a 11 pp (0.11, 95% CI 5.2, 17.0) increased likelihood of being in “very good” or “excellent” self-rated health associated with CA-PFL. o Reductions in psychological distress (-0.79, 95% CI -1.26, -0.32), the likelihood of being overweight (-0.082, 95% CI -0.150, -0.016), any alcohol consumption and daily consumption of three or more drinks (-0.120, 95% CI -0.160, -0.070) were also found for both birthing people and non-birthing partners. o The improvements in health status and psychological distress were greater for birthing people, and the reduction in alcohol use was greater for non-birthing partners.
8. Montoya-Williams et al. (2020) [26]	*n* = 6,164,203	California	1999–2008 / *Live birth and death certificates from all in-hospital deliveries from state vital statistics*	CA-PFL vs. two unexposed states (MO and PA)	Infant health outcomes: preterm birth (live birth occurring prior to 37 weeks), low birthweight (infant weighing < 2500 g), post-neonatal mortality (death occurring between 28 and 365 days of life), and overall infant mortality (death in the first year of life)	o Compared to unexposed states, CA-PFL was shown to have decreased post-neonatal death by 12% (*p* = 0.000). o There were no significant effects on the other health outcomes including pre-term birth, low birthweight, and infant mortality. o There were no differences in the effect by race/ethnicity or insurance status except for an increase in low birthweight among privately insured women in CA. o Reducing post-neonatal death by 1.94 per 10,000 live births was estimated to save 747 infant lives because of CA-PFL.
9. Morrissey et al. (2024) [27]	*n* = 136,525	New York	2011–2019 / *Behavioral Risk Factor Surveillance System (BRFSS)*	New York’s paid family leave (NYSPFL) vs. synthetic control	A self-reported binary measure of exercise in the past month and average hours of sleep per day (average 24-hour period)	o NYSPFL increased the likelihood of exercise among birthing people, single parents (0.11, 95% CI 0.048, 0.255), and low-income parents (0.072, 95% CI -0.052, 0.289). o Non-birthing partners, single parents, and those with two or more children showed an increase in daily sleep between 14 and 21 min per day. o There was no significant change in sleep for birthing people as a result of PFL.
10. Pihl & Basso (2019) [28]	*n* = 1,101,681	California	2000–2007 / *California Office of Statewide Health Planning and* *Development (OSHPD)*	CA-PFL vs. three non-PFL states (AZ, NY, WA)	Infant hospital admissions for lower respiratory, upper respiratory, skin infections, gastrointestinal infections, and cancer	o The introduction of CA-PFL showed a decline in all hospitalization among infants by 6% (-0.062, *p* = 0.017). o Specifically, infant hospital admissions for upper respiratory complications declined by 25% (-0.247, *p* = 0.078) and by 9% (-0.088, *p* = 0.034) for gastrointestinal infections. o There was no statistically significant change in infant hospital admissions for lower respiratory complications or cancer. o A 6% decrease in hospitalizations translated to 4,380 fewer hospitalizations.

Four of the included studies examined PFL’s association with infant health [20, 24, 26, 28], five investigated the association with parent health [21–23, 25, 27] and one looked at both parent and infant health outcomes [19].

Regarding the measures assessed for infant health, the post-neonatal mortality rate (PNMR) [20, 26], preterm birth, low birthweight, and infant mortality rates [26] were considered. Parent-reported overall infant health, infant food allergy, infant respiratory allergy [19], abusive head trauma (AHT) admissions [24], and hospital admissions for lower respiratory, upper respiratory, skin, and gastrointestinal infections, in addition to cancer were also reported [28].

Among the studies investigating parent health, two studies assessed physical health using self-reported health status on a five-point Likert scale, the number of nights spent in the hospital, the likelihood of reporting sick days, and daily prescription drug use [23]. Three studies examined postpartum psychological distress (PPD) using the Kessler Psychological Distress Scale (K6) [21, 22, 25]. Another looked at parent mental health on a five-point Likert scale [19]. Three studies reported well-being measures including self-reported exercise in the past month, self-reported number of hours slept in a 24-hour period [27], the likelihood of being overweight, and alcohol use [25].

## Synthesized findings

### Infant Health

#### Infant health – mortality rates

Two studies found improvements in the PNMR in the exposed state (CA for both) compared to unexposed states [20, 26]. They estimated PFL to be associated with a 12% annual decrease (*p* = 0.000) in PNMR, or approximately 750 lives saved per year [26], and an 8% decrease (*p* = 0.000) or approximately 340 lives saved per year [20].

#### Infant health – hospitalizations

Studies that examined infant hospitalizations also found improvements associated with CA-PFL. The policy was associated with a decrease of 5.11 in AHT admissions per 100,000 infants less than one year of age (-5.11, *p* = 0.035) [24]. In another study, PFL was associated with a 14.8 (-0.148, *p* = 0.045) and 24.7% (0.247, *p* = 0.078) decline in infant hospital admissions for gastrointestinal and upper respiratory infections, respectively [28]. Notably, this study suggested that a 6% decrease (-0.062, *p* = 0.017) in overall infant hospitalizations (across all conditions including skin and lower respiratory infections) translated to 4,380 fewer hospitalizations [28].

#### Infant health – other outcomes

Other improvements in infant health were found. Access to CA-PFL was associated with improved overall infant health by 7.4% points (0.074, *p* = 0.000), asthma by 2.7% points (-0.027, *p* = 0.013) and respiratory allergies by 2.4% points (-0.024, *p* = 0.004) relative to all non-PFL states, although no effect on infant food allergies was found (*p* = 0.090) [19]. The same study found no significant effect of CA-PFL on birthweight, preterm birth, or gestation length, in line with another included study that found no significant effect of CA-PFL on birthweight, preterm birth, or infant mortality [26].

### Parent health

Most studies found improvements for parent health in states exposed to PFL compared to unexposed states. Health outcomes were primarily analyzed across two groups (e.g. birthing people and non-birthing partners) and three primary categories (e.g. physical health, mental health, and well-being).

#### Parent health – physical health

Physical health was found to be improved for both birthing people and non-birthing partners in all studies, but the relative rates of improvement varied. Access to CA-PFL for birthing people was associated with a greater likelihood of being in “very good” or “excellent” self-rated health (0.243, *p* = 0.000) compared to non-birthing partners (0.149, *p* = 0.000) [25].

Birthing people with access to CA-PFL had reduced likelihoods of reporting sick days (-6.51, *p* = 0.000) compared to those without exposure to PFL as well as fewer nights in the hospital (-0.058, *p* = 0.000) and a lower likelihood of prescription drug use (-0.389, *p* = 0.000) [23]. Non-birthing partners also had a reduced likelihood of reporting sick days (-0.128, *p* = 0.000) but no change in the number of days spent in the hospital (0.455, *p* = 0.064) or the rate of prescription drug use (0.008, *p* = 0.699).

#### Parent health – mental health

Two of three studies found improvements in parent mental health in exposed states versus unexposed states. Most studies used the K6 scale, a 24-point scale. Access to PFL was found to be associated with a 0.636-point (95% CI = − 1.202, − 0.070) [17] and a 0.490-point (95% CI − 0.77, − 0.21) [18] improvement in mental health status for birthing people, and a 0.790-point improvement for birthing people and non-birthing partners together (95% CI -1.26, -0.32) [20]. The study that investigated parent mental health using a Likert scale found improvement in birthing person mental health status (0.086, *p* = 0.000), but no change in non-birthing partner mental health status (0.036, *p* = 0.421) in CA relative to non-PFL states [19]. To this end, mental health outcomes were inconclusive for non-birthing partners.

#### Parent health – well-being

Related to well-being, access to PFL for birthing people was associated with decreases in the likelihood of being overweight (-0.096, 95% CI=-0.19,-0.003) and small decreases in alcohol consumption (-0.017, 95% CI=-0.07,-0.04) [25]. Conversely, exposure to PFL for non-birthing partners was associated with notable decreases in alcohol consumption (-0.23, 95% CI=-0.29,-0.17) but no significant change in the likelihood of being overweight (-0.044, 95% CI =-0.10, 0.017) [25].These groups showed varying results related to exercise and sleep, and all results were insignificant [27].

### Parent health by subpopulation

Most of the included studies investigated outcomes for subpopulations of their samples, finding varying magnitudes of treatment effects in outcomes associated with PFL. Low-income households were more likely (0.179, *p* = 0.000) to report improvements in birthing person mental health than higher-income households (0.049, *p* = 0.000) (overall *p* = 0.000) [19]. Improvements in exercise and sleep were found to be significant for young, single and low-income parents – those less likely to have employer-provided paid leave – following the implementation of PFL in NY [27]. Relatedly, one study found that PFL was associated with greater benefits for birthing people who were Black or Hispanic relative to birthing people who were white compared to a synthetic control [21]; counter to these findings, another study found that PFL was associated with greater psychological distress for Black and Hispanic parents relative to white parents following the implementation of PFL in CA and NJ compared to non-PFL states [22].

### Risk of bias

Results of the ROBINS-I risk of bias assessment are summarized in Additional File 6, with tables categorized by outcome type and study. Risk of bias was evaluated separately for parent physical health and well-being outcomes [23, 25, 27], parent mental health outcomes [19, 21, 22, 25], and infant health physical health outcomes [19, 20, 24, 26, 28].

Among studies that examined parent physical health and well-being, two studies were rated as having serious risk of bias due to insufficient reporting of missing data [23, 27] and one was rated as having moderate risk of bias due to potential non-differential misclassification [25]. For parent mental health, one study was rated as serious risk of bias because of limited information on missing outcome data [19], and three studies were rated as moderate risk of bias due to possible non-differential misclassification of the intervention, as data sources did not report individual PFL use [21, 22, 25]. All studies on infant health outcomes were rated as having serious risk of bias due to potential misclassification of intervention status and lack of information on missing data. Overall, serious risk of bias most commonly stemmed from inadequate reporting of missing data. Moderate risk of bias was attributable to non-differential misclassification of the intervention.

## Discussion

This systematic review sought to evaluate how PFL is associated with the health outcomes of parents and infants in the U.S. All included studies found improvements in birthing people’s physical health, mental health, and well-being outcomes for those exposed to PFL compared to those not exposed. All studies found positive or neutral benefits of PFL for non-birthing partners’ and infants’ health outcomes. To our knowledge, this was the first systematic review of PFL’s association with health outcomes that was based on studies with quasi-experimental design for both parents and infants in the U.S.

### PFL’s association with parent and infant health outcomes

Exposure to PFL policies was associated with improvements in physical health, including a greater likelihood of improved self-rated health, reductions in number of nights spent in the hospital, the likelihood of reporting sick days, and daily prescription drug use for birthing people. In measures of mental health, PFL was associated with improvements in PPD, and parent mental health. Related to well-being, those exposed to PFL showed a decrease in alcohol consumption as well as the likelihood of being overweight.

Studies reported positive and neutral associations between PFL and non-birthing partners’ health outcomes. PFL exposure was associated with increases in self-reported health and reductions in the likelihood of reporting sick days, although it was not associated with any changes in the number of days spent in hospital or prescription drug use. Mental health outcomes were contradictory: two studies found PFL not to be associated with any change in non-birthing partners’ mental health status, while another found improvements. On well-being measures, PFL access was associated with notable decreases in alcohol consumption for non-birthing partners.

All studies that examined infant health outcomes found that PFL was associated with positive or neutral effects. Exposure to PFL was associated with improvements in the PNMR, overall parent-reported infant health, infant hospitalizations related to AHT admissions, upper respiratory infections, and gastrointestinal complications. PFL was found to be associated with neutral effects on food and respiratory allergies, low birthweight, preterm birth, gestation length, and infant mortality.

### Implications

Counter to several studies’ findings [19, 21, 29] which reported that PFL held the greatest benefits for parents who were low-income, single, young, Black or Hispanic, U.S.-born, and of lower educational status in CA, Irish and colleagues reported a widening racial disparity in mental health outcomes following the implementation of CA and NJ state-wide PFL policies. A potential reason for this discrepancy is the differential uptake of state-sponsored leave across social identities [3]. While all studies evaluated health impacts related to access to PFL, this measure does not ensure that those with access use that leave, leading to inaccurate estimates of PFL’s impact on different subpopulations. Without attention to the difference between access and use of PFL, the association of this policy with health outcomes may be underestimated, and consequentially, the inequitable health outcomes associated with barriers to use of PFL may not be detected or improved.

Another factor with potential implications for this review’s findings is the variation in employer-provided paid leave policies, which are present in all states, even comparison states without PFL policies. Employer-provided paid leave and other traditional socioeconomic policies have been shown to reify systematic inequities, buffering the potential equity-driven impacts of state-sponsored PFL policies [30]. Additionally, heterogeneity in the design of state-sponsored PFL policies may influence findings. Although most studies investigated the effects of CA-PFL, policies in NY and NJ were also evaluated. These policies vary in duration of implementation, level of job protection, wage replacement, and other key design features. These factors may impact health outcomes, although more research is needed on this topic. Future implementation efforts should prioritize increasing uptake among racially minoritized and low-income groups, who experience lower rates of PFL use and face pre-existing inequities in health outcomes [3].

Three included studies’ results diverged on PFL’s association with non-birthing partners’ mental health status. Inconsistencies in these findings may be associated with the amount of leave taken by non-birthing partners. Studies show that non-birthing partners are less likely to take full leave compared to birthing people [31, 32]. More research is needed to understand the health outcomes associated with PFL following childbirth for non-birthing partners as well as how PFL use for non-birthing partners may be associated with birthing people’s health outcomes.

### Limitations

It is important to note the limitations of this review. First, this review investigated a small body of literature given its focus on natural experiments. As such, analyzing trends across health outcomes and multiple studies was difficult. Second, this review was limited in its geographic setting: eight of the ten included studies used CA as the exposed state. Most of the results in this review apply only to CA. Third, all studies assessed outcomes only within the first year postpartum, limiting the understanding of the long-term effects of PFL. Fourth, this review was not registered on PROSPERO prior to its conduct.

Related to individual studies, several included studies relied on self-reported or parent-reported measures, which are subject to response bias. Finally, it is also worth mentioning that all included studies were rated as having either serious or moderate risk of bias. Serious risk of bias was typically due to insufficient detail about missing outcome data, while moderate risk was attributable to non-differential misclassification, as several studies estimated intention-to-treat effects using state-level PFL access rather than individual-level policy use.

### Future directions

Many of the included studies in this review point to the need for future research that examines the association between PFL and health disparities across birthing people, non-birthing partners, and infants. Given the inconclusive findings on parent mental health associated with PFL across racial and ethnic subgroups, future work would benefit from investigating why there are differences in outcomes associated with PFL and how policies can be tailored to benefit marginalized groups. These are essential considerations as unjust cycles of disparate health outcomes persist among those who are less likely to have access to PFL. Future work would benefit from a greater diversity of analysis across participant social characteristics, such as education status, insurance status, gender identity, immigration status, ability, religion, etc., with attention to intersectional identities.

Related to policy design, future quasi-experimental work should consider examining how the generosity of PFL policies impacts health outcomes. PFL policies differ in many characteristics such as duration, level of job protection, and percent of wages replaced; cumulatively, these characteristics are likely to moderate the policies’ health effects. As additional states implement PFL, future research should assess how differences in policy design affect health outcomes.

Research conducted outside of the postpartum period may be beneficial for a more holistic understanding of PFL’s association with health outcomes. More work on PFL’s association with long-term benefits for children beyond their first years of life would be useful [33]. Relatedly, long-term benefits to parents could strengthen PFL’s robustness as a preventative measure against negative health outcomes later in life.

Finally, outside of individual-level indicators of health, studies that investigate population level indicators of birthing people’s health outcomes may be insightful on the benefits of PFL. Useful indicators are the maternal mortality ratio (MMR) and severe maternal morbidity (SMM), pregnancy outcomes which can result in significant short- and long-term health consequences [34]. Investigating the association between PFL and these health measures would provide greater understanding into its capacity to improve health equity, as MMR and SMM have been important indicators for assessing racial birth equity [35]. A study of this nature could serve as a step toward addressing the maternal health crisis in the U.S.

## Conclusions

This work synthesized a small body of literature focused on the association between PFL and parent and infant health outcomes. All included studies found PFL to be associated with improvements in birthing people’s physical health, mental health, and well-being outcomes for those exposed to PFL compared to those not exposed. Studies found PFL to be associated with positive or neutral effects for non-birthing partners and infants. This study was among the first to systematically review studies with quasi-experimental designs on PFL’s association with health outcomes and comparability across studies. It synthesized differential treatment effects associated with PFL for marginalized groups and is a step toward understanding health inequities associated with the absence of state-sponsored PFL policies. It offered future directions that include research on health inequities with intersectional analysis. A more comprehensive understanding of PFL’s effects on parents’ and infants’ health outcomes could inform implementation strategies that promote a more equitable distribution of PFL’s health benefits.

## Supplementary Information

Additional File 1: Paid Family Leave Policy Characteristics by State. The table outlines various characteristics of PFL policies by state, including job protection, wage replacement, maximum weekly amount, etc.

Additional File 2: Search Strategy. Example of PubMed search strategy used for this review.

Additional File 3: PRISMA-ScR Checklist. A checklist detailing the pages of the review in which the criteria for the PRISMA-ScR are satisfied.

Additional File 4: Completed Data Abstraction Table. The data abstraction table was used to abstract data from each included study. This file includes the completed tables for all included studies in this review.

Additional File 5: PRISMA Diagram. PRISMA diagram outlining the results of the study screening process.

Additional File 6: ROBINS-I Results. Results of ROBINS-I risk assessment by outcome type including parent physical health, parent mental health and infant physical health.

## Data Availability

No datasets were generated or analysed during the current study.

## Abbreviations

AHT: Abusive head trauma
CA-PFL: California Paid Family Leave
K6: Kessler Psychological Distress Scale
MMR: Maternal mortality ratio
PFL: Paid Family Leave
PNMR: Post-Neonatal Mortality Rate
SMM: Severe maternal morbidity
U.S.: United States
