# Supplementary material for: The impact of paid family leave on parent and infant health outcomes: a systematic review of quasi-experimental research
Source: BMC Public Health. 2026 Jul 14;26:2914. doi: 10.1186/s12889-026-27709-2 (PMC13651160; doi:10.1186/s12889-026-27709-2)
Supplement: Supplementary file 2 [file 12889_2026_27709_MOESM1_ESM.docx]

Paid Family Leave Policy Characteristics by State

| **State** | **Date Enacted** | **Date Effective** | **Job Protection** | **Wage Replace-ment** | **Maximum Weekly Amount (2026)** | **Total PFL Premium as Share of Employee Wages** | **Maximum Weeks of Paid Leave** |
| --- | --- | --- | --- | --- | --- | --- | --- |
| CA [(1,2)](https://sciwheel.com/work/citation?ids=18463116,18664986&pre=&pre=&suf=&suf=&sa=0,0&dbf=0&dbf=0) | 2002 | 2004 | No | 90% | $1,765 | 1.30% | 8 |
| CO [(1,3)](https://sciwheel.com/work/citation?ids=18463116,18664956&pre=&pre=&suf=&suf=&sa=0,0&dbf=0&dbf=0) | 2020 | 2024 | Yes* | 90% | $1,382 | 0.88% | 12 |
| CT [(1,4)](https://sciwheel.com/work/citation?ids=18463116,17669350&pre=&pre=&suf=&suf=&sa=0,0&dbf=0&dbf=0) | 2019 | 2022 | No | 95% | $1,016 | 0.50% | 12 |
| DE [(1,5)](https://sciwheel.com/work/citation?ids=18463116,18664962&pre=&pre=&suf=&suf=&sa=0,0&dbf=0&dbf=0) | 2022 | 2026 | Yes* | 80% | $900 | 0.80% | 12 |
| D.C. [(1,6)](https://sciwheel.com/work/citation?ids=18463116,18462643&pre=&pre=&suf=&suf=&sa=0,0&dbf=0&dbf=0) | 2017 | 2020 | No | 90% | $1,190 | 0.75% | 12 |
| ME [(1,7)](https://sciwheel.com/work/citation?ids=18463116,18664964&pre=&pre=&suf=&suf=&sa=0,0&dbf=0&dbf=0) | 2023 | 2026 | Yes | 90% | $1,198 | 1.00% | 12 |
| MD [(1,8)](https://sciwheel.com/work/citation?ids=18463116,18664969&pre=&pre=&suf=&suf=&sa=0,0&dbf=0&dbf=0) | 2022 | 2026 | Yes | 90% | $1,000 | 0.90% | 12 |
| MA [(1,9)](https://sciwheel.com/work/citation?ids=18463116,18462629&pre=&pre=&suf=&suf=&sa=0,0&dbf=0&dbf=0) | 2018 | 2021 | Yes | 80% | $1,230 | 0.46% | 12 |
| MN [(1,10)](https://sciwheel.com/work/citation?ids=18463116,18664972&pre=&pre=&suf=&suf=&sa=0,0&dbf=0&dbf=0) | 2023 | 2026 | Yes | 90% | $1,423 | 0.44% | 12 |
| NJ [(1,11)](https://sciwheel.com/work/citation?ids=18463116,18664975&pre=&pre=&suf=&suf=&sa=0,0&dbf=0&dbf=0) | 2008 | 2009 | No | 85% | $1,119 | 0.23% | 12 |
| NY [(1,12)](https://sciwheel.com/work/citation?ids=18463116,17995499&pre=&pre=&suf=&suf=&sa=0,0&dbf=0&dbf=0) | 2016 | 2018 | Yes | 67% | $1,229 | 0.43% | 12 |
| OR [(1,13,14)](https://sciwheel.com/work/citation?ids=18463116,18462627,18462653&pre=&pre=&pre=&suf=&suf=&suf=&sa=0,0,0&dbf=0&dbf=0&dbf=0) | 2019 | 2023 | Yes* | 100% | $1,637 | 1.00% | 12 |
| RI [(1,15)](https://sciwheel.com/work/citation?ids=18463116,18664979&pre=&pre=&suf=&suf=&sa=0,0&dbf=0&dbf=0) | 2013 | 2014 | Yes | 60% | $1,103 | 1.10% | 6 |
| WA [(1,16)](https://sciwheel.com/work/citation?ids=18463116,18462608&pre=&pre=&suf=&suf=&sa=0,0&dbf=0&dbf=0) | 2017 | 2020 | Yes | 90% | $1,647 | 1.13% | 12 |

The date enacted is when the policy became law, and the date effective is when the policy became active. Job protection refers to legal rights in which employees can take state-sponsored leave without losing their jobs. Wage replacement is the percentage of employees’ wages which will be replaced under the policy; however, each state has a maximum weekly amount, which cannot be exceeded regardless of employee income. Total PFL premium as share of employee wages is the percentage of employees’ incomes which are used to fund state leave programs. Finally, maximum weeks of paid leave are the number of weeks sponsored by the policy as time away from work.

*Job protection in these states applies to employees who have been with their employer for at least 180 (CO), 120 (DE) and 90 (OR) days.

References

[1.    Bipartisan Policy Center. State Paid Family Leave Laws Across the U.S. [Internet]. 2025 [cited 2025 Dec 9]. Available from: https://bipartisanpolicy.org/explainer/state-paid-family-leave-laws-across-the-u-s/](https://sciwheel.com/work/bibliography/18463116)

[2.    California Employment Development California. Paid Family Leave [Internet]. 2026 [cited 2026 Feb 6]. Available from: https://edd.ca.gov/en/disability/paid-family-leave/](https://sciwheel.com/work/bibliography/18664986)

[3.    Colorado Family and Medical Insurance Program [Internet]. 2026 [cited 2026 Feb 5]. Available from: https://famli.colorado.gov/](https://sciwheel.com/work/bibliography/18664956)

[4.    Connecticut Paid Leave. Connecticut Paid Leave [Internet]. 2025 [cited 2025 Mar 22]. Available from: https://www.ctpaidleave.org/?language=en_US](https://sciwheel.com/work/bibliography/17669350)

[5.    Delaware Department of Labor. Delaware Paid Leave [Internet]. 2025 [cited 2026 Feb 5]. Available from: https://labor.delaware.gov/delaware-paid-leave/](https://sciwheel.com/work/bibliography/18664962)

[6.    DC Paid Family Leave. Department of Employment Services; 2020 Jun.](https://sciwheel.com/work/bibliography/18462643)

[7.    State of Maine Department of Labor. Paid Family and Medical Leave [Internet]. 2025 [cited 2026 Feb 19]. Available from: https://www.maine.gov/paidleave/](https://sciwheel.com/work/bibliography/18664964)

[8.    Maryland Family and Medical Leave Insurance. Paid family and medical leave is coming to Maryland [Internet]. 2025 [cited 2026 Feb 5]. Available from: https://paidleave.maryland.gov/](https://sciwheel.com/work/bibliography/18664969)

[9.    Alpine WJ. FY2025 Annual Report for the Massachusetts Paid Family and Medical Leave Program . The Commonwealth of Massachusetts; 2025.](https://sciwheel.com/work/bibliography/18462629)

[10.   Minnesota Paid Leave. About Paid Leave [Internet]. 2025 [cited 2026 Feb 5]. Available from: https://paidleave.mn.gov/](https://sciwheel.com/work/bibliography/18664972)

[11.   New Jersey Department of Labor & Workforce Development. Family Leave Insurance [Internet]. 2025 [cited 2026 Feb 5]. Available from: https://www.nj.gov/labor/myleavebenefits/worker/fli/](https://sciwheel.com/work/bibliography/18664975)

[12.   New York State. New York Paid Family Leave Updates for 2025 [Internet]. 2025 [cited 2025 Jun 24]. Available from: https://paidfamilyleave.ny.gov/2025](https://sciwheel.com/work/bibliography/17995499)

[13.   Paid Leave Oregon: Benefits. Employment Department; 2025.](https://sciwheel.com/work/bibliography/18462627)

[14.   Bureau of Labor and Industries. Oregon Family Leave Act [Internet]. Bureau of Labor and Industries. [cited 2025 Dec 9]. Available from: https://www.oregon.gov/boli/workers/pages/oregon-family-leave.aspx](https://sciwheel.com/work/bibliography/18462653)

[15.   Rhode Island Paid Leave. Paid Leave [Internet]. 2025 [cited 2026 Feb 5]. Available from: https://ripaidleave.net/](https://sciwheel.com/work/bibliography/18664979)

[16.   DeFour J. Paid Family & Medical Leave Report . Employment Security Department; 2023 Dec.](https://sciwheel.com/work/bibliography/18462608)
