# Supplementary material for: The impact of paid family leave on parent and infant health outcomes: a systematic review of quasi-experimental research
Source: BMC Public Health. 2026 Jul 14;26:2914. doi: 10.1186/s12889-026-27709-2 (PMC13651160; doi:10.1186/s12889-026-27709-2)
Supplement: Supplementary file 3 [file 12889_2026_27709_MOESM2_ESM.docx]

Search Strategy

Date of Search – March 1, 2024

PubMed Search Strategy

| **Search topic** | **Search terms** | **Number of articles** |
| --- | --- | --- |
| Paid Family Leave | ("paid leave"[tw] OR "parental leave"[tw] OR "parental leaves"[tw] OR "family leave"[tw] OR "family leaves"[tiab] OR "maternity leave"[tw] OR "maternity leaves"[tw] OR "paternity leave"[tw] OR "paternity leaves"[tiab] OR "maternal leave"[tw] OR "maternal leaves"[tiab] OR "paternal leave"[tw] OR "paternal leaves"[tiab] OR "pregnancy leave"[tw] OR "pregnancy leaves"[tiab] OR "antenatal leave"[tw] OR "antenatal leaves"[tiab] OR "prenatal leave"[tw] OR "prenatal leaves"[tiab] OR "postpartum leave"[tiab] OR "postpartum leaves"[tiab] OR "workplace leave"[tiab] OR "bonding leave"[tiab] OR "adoption leave"[tiab] OR "fostering leave"[tiab] OR "paid time off"[tw] OR "temporary leave"[tw] OR "disability leave"[tw] OR "temporary disability leave"[tiab] OR "caregiving leave"[tw] OR "unpaid leave"[tw] OR "medical leave"[tw] OR "sick leave"[tw] OR "annual leave"[tw] OR "bereavement leave"[tw])  AND  (paid[tw] OR job-protected[tw] OR "job protected"[tw] OR employer-paid[tw] OR "employer sponsored"[tw] OR employer-sponsored[tw] OR state-sponsored[tw] OR "state sponsored"[tw] OR "state provided"[tw] OR "wage replacement"[tw] OR "partial wage replacement"[tiab] OR compensated[tw] OR pay[tw] OR payment[tw] OR compensation[tw] OR compensate[tw] OR protection[tw] OR protected[tw] OR sponsored[tw] OR unpaid[tw] OR uncompensated[tw] OR guaranteed[tw] OR salary[tw] OR subsidize[tw] OR subsidized[tw] OR subsidy[tw])  AND  (infant[tw] OR baby[tw] OR child[tw] OR family[tw] OR mother[tw] OR mothers[tw] OR father[tw] OR fathers[tw] OR parent[tw] OR parents[tw] OR maternity[tw] OR paternity[tw] OR childbirth[tw] OR pregnant[tw] OR pregnancy[tw] OR postpartum[tw] OR adopt[tw] OR adoption[tw] OR breastfeeding[tw] OR lactation[tw] OR breastfeed[tw] OR lactating[tw] OR "foster care"[tw] OR "foster parent"[tw] OR "foster parenting"[tw])  NOT  ("systematic review"[pt] OR comment[pt] OR letter[pt]) | 939 |
