# Supplementary material for: The impact of paid family leave on parent and infant health outcomes: a systematic review of quasi-experimental research
Source: BMC Public Health. 2026 Jul 14;26:2914. doi: 10.1186/s12889-026-27709-2 (PMC13651160; doi:10.1186/s12889-026-27709-2)
Supplement: Supplementary file 4 [file 12889_2026_27709_MOESM4_ESM.docx]

**Completed Data Abstraction Tables**

1. Bullinger (2019). “The Effect of Paid Family Leave on Infant and Parental Health in the United States.”
2. Chen *et al.* (2023). “Does Paid Family Leave Save Infant Lives? Evidence From California's Paid Family Leave Program.”
3. Doran *et al.* (2020). “California's Paid Family Leave Law Improves Maternal Psychological Health.”
4. Irish *et al.* (2021). “Paid Family Leave And Mental Health In The U.S.: A Quasi-Experimental Study Of State Policies.”
5. Kim (2024). “The Effects Of Paid Family Leave—Does It Help Fathers’ Health, Too?”
6. Klevens *et al.* (2017). “Paid Family Leave's Effect On Hospital Admissions For Pediatric Abusive Head Trauma.”
7. Lee *et al.* (2020). “The Effect Of California's Paid Family Leave Policy On Parent Health: A Quasi-Experimental Study.”
8. Montoya-Williams *et al.* (2020). “The Impact Of Paid Family Leave In The United States On Birth Outcomes And Mortality In The First Year Of Life.”
9. Morrissey *et al.* (2024). “The Impacts of New York State's Paid Family Leave Policy on Parents' Sleep and Exercise.”
10. Pihl & Basso (2019). “Did California Paid Family Leave Impact Infant Health?”

**Bullinger (2019). “The Effect of Paid Family Leave on Infant and Parental Health in the United States.”**

| **Citation Details** | Author(s) | Lindsey Rose Bullinger |
| --- | --- | --- |
|  | Name of Article | “The Effect of Paid Family Leave on Infant and Parental Health in the United States.” |
|  | Author Affiliation | School of Public Policy, Georgia Tech |
|  | Journal Name | *Journal of Health Economics* |
|  | Date of Publication | 2019 |
|  | Funding Source | N/A |
| **Study Aims** |  | This study aims to estimate the impact of California’s paid family leave program on the health and well-being of infants and their parents. |
| **Population** | Total sample size (n) | n = 22,050 |
|  | Age | N/A |
|  | Gender of participants | Female only |
|  | Race / Ethnicity | N/A |
|  | Income | N/A |
|  | Insurance (e.g., Private, Medicaid, no insurance) | N/A |
|  | Education Status | N/A |
|  | Other Relevant Characteristics Tracked | Birth weight, gestational age, prenatal care, smoking |
|  | Eligibility Criteria (e.g. inclusion / exclusion criteria) | Inclusion: Infants aged 0 to 1 year and their parents  Exclusion: Infants aged 2 to 3 years and their parents |
| **Intervention:** Paid Parental / Family Leave | Type of Leave | California Paid Family Leave (CA-PFL) |
|  | Duration of Leave | 6 weeks |
|  | Pay While on Leave | 55% wage replacement |
|  | Comparison Groups | CA-PFL vs. non-PFL neighboring states (Control 1 = NV, AZ, WA), non-PFL big states (Control 2 = TX, FL, NY, PA), all non‐CA states plus D.C. (Control 3) |
| **Outcome:**  Parent and Infant Health Outcomes | Health Indicator(s) measured | Parent-reported overall infant health, infant food allergy, infant respiratory allergy, and parent mental health |
|  | Secondary Indicator(s) | N/A |
|  | Method of measurement (e.g. self-report, vital statistics, etc.) | Vital statistics |
| **Study Design** | Type of Quasi-Experimental Design | Difference-in-differences (DiD) |
|  | Additional details | N/A |
| **Data Source** | Type | National Survey of Children’s Health (NSCH) and Behavioral Risk Factor Surveillance System (BRFSS) |
|  | Years of data source | 2003, 2007, 2011 – 2012 (NSCH) and 2001 – 2007 (BRFSS) |
|  | Location(s) | California and other U.S. states |
| **Methods** | Data Analysis | Difference-in-differences analysis |
|  | Covariates | Maternal age, race/ethnicity, education, marital status, maternal employment status |
| **Main Findings** | Main Findings | - Access to CA-PFL improved overall child health by 9 percentage points (pp) (0.086, p=0.000) relative to neighboring states, 5 pp (0.048, p=0.169) relative to other large states, and 7 pp (0.074, p=0.000) relative to all non-PFL states. - CA-PFL was suggested to improve birthing person mental health status by up to 18 pp (0.177,p=0.002) relative to other large states. - Non-birthing partners were not found to experience any improvements in mental health status due to PFL. - Due to CA-PFL, low-income households were more likely to report improvements in maternal mental health (p=0.000). - Infants showed a reduction in asthma by 2.7 pp (-0.027, p=0.013) relative to neighboring states and all non-PFL states. - There were no significant effects of PFL on respiratory allergies or food allergies. - CA-PFL had no effect on low-birthweight, preterm birth, or gestation length. |
|  | Other findings (e.g. cost, implementation, feasibility, dissemination) | N/A |
|  | Implications of Research on Policy or Practice | - The findings suggest that CA-PFL improved both child and parental health outcomes, indicating that PFL can be an effective public health tool. - Because the study did not directly measure leave-taking, the results imply that increasing awareness, eligibility, and uptake of PFL could further enhance health benefits. |
| **Conclusions** |  | CA-PFL improved infant health and maternal health behaviors. |
| **Future Directions** |  | - The study relied heavily on **parent-reported health outcomes,** so future research should use clinical or administrative health data to validate findings. - The study focused on relatively short-term outcomes (e.g., early child health, parental mental health). Future studies should examine longer-term impacts on child development and family well-being. |
| **Strengths / Limitations** | Strengths of methods | - The use of multiple comparison groups to validate findings strengthens confidence that findings are not driven by a single comparison. - Results are consistent across different model specifications and robustness checks. |
|  | Limitation of methods | - Multiple outcomes (e.g., overall infant health, and parent mental health) are reported by parents, which may have introduced reporting bias - The data source did not observe leave-taking, meaning treatment in this study was exposure to PFL as opposed to use of PFL. - Findings are based on California’s specific policy context and may not generalize to other states. |

**Chen (2023). “Does Paid Family Leave Save Infant Lives? Evidence From California's Paid Family Leave Program.”**

| **Citation Details** | Author(s) | Feng Chen |
| --- | --- | --- |
|  | Name of Article | “Does Paid Family Leave Save Infant Lives? Evidence From California's Paid Family Leave Program.” |
|  | Author Affiliation | Institute of Economic Research, Liaoning University |
|  | Journal Name | *Contemporary Economic Policy* |
|  | Date of Publication | 2023 |
|  | Funding Source | N/A |
| **Study Aims** |  | - This paper examined the overall effect of California’s paid family leave policy on the post-neonatal mortality rate in the U.S. - It also assessed the heterogeneous effects of PFL for different subgroups of mothers and infants. |
| **Population** | Total sample size (n) | n = 5,508 |
|  | Age | < 20 years: 0.16, 20-25 years: 0.26, 25-30 years: 0.27, 30-35 years: 0.21, >35 years: 0.11 |
|  | Race / Ethnicity | Black: 0.14, White: 0.56, Other: 0.06, Hispanic: 0.24 |
|  | Income | Average: $47,950 |
|  | Insurance (e.g., Private, Medicaid, no insurance) | N/A |
|  | Education Status | <High School: 0.17, High School Grad: 0.28, Some College: 0.26, College or More: 0.29 |
|  | Other Relevant Characteristics Tracked | Fertility rate |
|  | Eligibility Criteria (e.g. inclusion / exclusion criteria) | Inclusion: All singleton births and infant deaths from 2000 to 2008  Exclusion: Multiple births |
| **Intervention:** Paid Parental / Family Leave | Type of Leave | California Paid Family Leave (CA-PFL) |
|  | Duration of Leave | 6 weeks |
|  | Pay While on Leave | 55% wage replacement, up to a weekly cap of $728 in 2004 |
|  | Comparison Groups | CA vs. all non‐CA states plus D.C. |
| **Outcome:**  Parent and Infant Health Outcomes | Health Indicator(s) measured | Post-neonatal mortality rate [PNMR] (defined as infant deaths between 28 and 365 days per 1000 live births) |
|  | Secondary Indicator(s) | N/A |
|  | Method of measurement (e.g. self-report, vital statistics, etc.) | Vital statistics |
| **Study Design** | Study Design | Quasi-experimental |
|  | Additional details | N/A |
| **Data Source** | Type | National Vital Statistics Survey |
|  | Years of data source | 2000 – 2008 |
|  | Location(s) | CA vs. all other states |
| **Methods** | Data Analysis | Difference-in-difference |
|  | Covariates | The birth controls are birth weight, gestational age, sex at birth, and birth order. The maternal controls are age, race/ethnicity, marital status, educational attainment, employment status, and family income. |
| **Main Findings** | Main Findings | - PFL reduced PNMR by 0.135 (p=0.000), or approximately 339 infant lives. - There were fewer deaths from health‐related causes and larger effects for infants with married mothers and for infant boys. |
|  | Other Findings (e.g. cost, implementation, feasibility, dissemination) | - The decline in PNMR was larger among infants born to married mothers, potentially reflecting greater ability to take advantage of leave benefits. - The study finds smaller or non-significant effects among infants born to disadvantaged mothers (e.g., unmarried or lower socioeconomic status), suggesting barriers to access or uptake of PFL. - Infants of more educated mothers tended to experience stronger mortality reductions, pointing to differential access or ability to utilize paid leave. |
|  | Implications of Research on Policy or Practice | - The benefits of the PFL program may be understated, which is especially significant as more states in the U.S. develop their own PFL programs. - If a 12-week national PFL program took effect, PNMR would be 0.27, and in 2020 with 3.6 million new births, this would save approximately 972 additional infants. |
| **Conclusions** |  | - This study examined the first PFL program in the U.S. and found that a 6‐week PFL reduced PNMR by 0.135, saving approximately 339 infant lives in CA from 2004 to 2008. - Heterogeneous analyses suggested that there were fewer deaths from health‐related causes and larger effects for infants with married mothers and for infant boys. |
| **Future Directions** |  | - Future work would benefit from investigation into why differences in outcomes exist and whether policies can be tailored to benefit marginalized populations. - Additional research could evaluate long-term child health and developmental outcomes beyond infant mortality, as well as impacts on family well-being. - Future studies should examine whether similar effects occur in other states or under different policy designs (e.g., more generous leave, longer duration). |
| **Strengths / Limitations** | Strengths of methods | - The study used **linked birth and infant death records,** allowing for direct measurement of infant mortality rather than relying only on surviving infants. - The difference-in-differences approach compared CA to other states over time, helping to isolate the causal effect of PFL. - The author conducted multiple robustness checks to demonstrate that results were unlikely driven by confounding factors or contemporaneous shocks. |
|  | Limitation of methods | - The analysis measured policy exposure rather than actual uptake of the policy, which may lead to an underestimation of effects. - There are additionally limited insight into the mechanisms of why PFL is associated with a decrease in mortality. - Findings are based on CA;s specific policy context, which may not generalize to other states with different leave structures. |

**Doran et al (2020). “California's Paid Family Leave Law Improves Maternal Psychological Health.”**

| **Citation Details** | Author(s) | Elizabeth L. Doran,^a^ Ann P. Bartel,^b^ Christopher J. Ruhm,^c^ Jane Waldfogel^a^ |
| --- | --- | --- |
|  | Name of Article | “California's Paid Family Leave Law Improves Maternal Psychological Health.” |
|  | Author Affiliation | ^a^ Columbia University School of Social Work  ^b^ Columbia Business School  ^c^ Frank Batten School of Leadership and Public Policy University of Virginia |
|  | Journal Name | *Social Science & Medicine* |
|  | Date of Publication | 2020 |
|  | Funding Source | Robert Wood Johnson Foundation Policies for Action grant #RWJ 74030  National Institute for Child Health and Human Development grant P2CHD058486 |
| **Study Aims** |  | To study the effect of California's first in the nation paid family leave policy on maternal postpartum psychological distress for women overall and for disadvantaged groups. |
| **Population** | Total sample size (n) | n = 7,379  The primary analysis sample contained 1652 California mothers with infants (726 before and 926 after CA-PFL) and 5,727 mothers with infants in a weighted average of 35 other states constituting the synthetic control group (2,215 before and 3,512 after CA-PFL). |
|  | Age | Pre-Law: 45.7% 18 – 29 years, 54.3% 30+ years  Post-Law: 45.3% 18 – 29 years, 54.8% 30+ years |
|  | Gender of participants | Pre-Law 50.0% Female, 50.0% Male  Post-Law 49.4% Female, 50.6% Male |
|  | Race / Ethnicity | Pre-Law: 4.7% Black non-Hispanic, 52.4% Hispanic, 9% Other, 33.9% White non-Hispanic  Post-Law: 7.2% Black non-Hispanic, 50.3% Hispanic, 10.5%% Other, 32% White non-Hispanic |
|  | Income | N/A |
|  | Insurance (e.g., Private, Medicaid, no insurance) | N/A |
|  | Education Status | Pre-Law: 37.3% High School or Less, 35.7% Some College, 27.1% College Grad  Post-Law: 28.8% High school or Less, 41.2% Some College, 30.0% College Grad |
|  | Other Relevant Characteristics Tracked | Pre-Law: 52.7% Employed, 47.3% Not Employed  Post-Law: 55.8% Employed, 44.2% Not Employed  Place of birth, marital status, parity |
|  | Eligibility Criteria (e.g. inclusion / exclusion criteria) | Inclusion: Mothers with infants (under 12 months old) in each NHIS annual survey wave from 2000 to 2010 without missing information on the child's birthdate |
| **Intervention:** Paid Parental / Family Leave | Type of Leave | California Paid Family Leave (CA-PFL) |
|  | Duration of Leave | 6 weeks |
|  | Pay While on Leave | 55% wage replacement |
|  | Comparison Groups | CA versus “Synthetic California” (Abadie et al., 2010; Abadie; Cattaneo, 2018). |
| **Outcome:**  Parent and Infant Health Outcomes | Health Indicator(s) measured | Their measure of maternal postpartum psychological distress (PPD) was obtained from the six-item Kessler Psychological Distress Scale (K6) across marital status, age, race/ethnicity, and education |
|  | Secondary Indicator(s) | N/A |
|  | Method of measurement (e.g. self-report, vital statistics, etc.) | Self-report |
| **Study Design** | Type of Quasi-Experimental Design | Difference-in-difference with synthetic control |
|  | Additional details | This study provided the first quasi-experimental evidence on the impact of a state PFL law on maternal postpartum psychological distress in the U.S. using a rigorously validated measure of psychological health. |
| **Data Source** | Type | National Health Interview Survey |
|  | Years of data source | 2000 to 2010 |
|  | Location(s) | California |
| **Methods** | Data Analysis | - Differences-in-differences (DD) analysis - They used OLS with an interaction between CA and post-treatment to generate the treatment effect for the continuous K6 score |
|  | Covariates | - They included controls for maternal and child demographic characteristics in constructing their Synthetic California control group. - Maternal controls included race/ethnicity, education, age at child's birth, marital status, and country of birth. - The child controls were age, birth order, and gender. They excluded income as a control because it is likely to be directly influenced by leave-taking. |
| **Main Findings** | Main Findings | - Access to PFL was associated with a 0.636-point decrease point (95% CI = −1.202, −0.070) in PPD symptoms among birthing people with infants. - CA-PFL was found to hold more benefits for Black and Hispanic birthing people (compared to White birthing people) and non-college-educated birthing people (compared to college-educated birthing people) - Birthing people who were young, single, and U.S.-born, were shown to benefit more from CA-PFL than their counterparts. |
|  | Other findings (e.g. cost, implementation, feasibility, dissemination) | Results showed that the effect of CA-PFL was highly concentrated among mothers with mild rather than moderate PPD. |
|  | Implications of Research on Policy or Practice | The findings from this study underscored the importance of paid time with newborns as a protective factor against mild maternal PPD. |
| **Conclusions** |  | PFL was associated with improved mental health for California mothers, suggesting that expansions of state or federal PFL policies have the potential to improve maternal postpartum health. |
| **Future Directions** |  | - Future research focused on the health impacts of PFL as opposed to labor market impacts would be beneficial. |
| **Strengths / Limitations** | Strengths of methods | - The use of synthetic control to create a quasi-experimental design - The use of the Kessler Psychological Distress Scale: (K6), a rigorously validated measure of psychological health. |
|  | Limitation of methods | - Their subgroup analysis had low precision due to small sample sizes. - It is possible that their primary outcome measure, the K6 score, is biased by measurement error, as cultural and linguistic differences for marginalized populations may result in misclassification of psychological distress. |

**Irish et al (2021). “Paid Family Leave and Mental Health in the U.S.: A Quasi-Experimental Study of State Policies.”**

| **Citation Details** | Author(s) | Amanda M. Irish, DVM, MPH,^a^ Justin S. White, PhD,^a,b^ Sepideh Modrek, PhD,^c^ Rita Hamad, MD, PhD^b,d^ |
| --- | --- | --- |
|  | Name of Article | “Paid Family Leave and Mental Health in the U.S.: A Quasi-Experimental Study of State Policies.” |
|  | Author Affiliation | ^a^ Department of Epidemiology & Biostatistics, University of California San Francisco  ^b^ Philip R. Lee Institute for Health Policy Studies, University of California San Francisco  ^c^ Health Equity Institute, San Francisco State University  ^d^ Department of Family and Community Medicine, University of California San Francisco |
|  | Journal Name | *American Journal of Preventive Medicine* |
|  | Date of Publication | 2021 |
|  | Funding Source | University of California San Francisco National Center for Excellence in Women’s Health |
| **Study Aims** |  | This study aimed to test the hypothesis that paid family leave policies in California and New Jersey improved parent mental health. |
| **Population** | Total sample size (n) | n = 28,638 |
|  | Age | CA: 31.1 years, NJ: 32.3 years, Other states: 30.2 years |
|  | Gender of participants | CA: 56% Female, NJ: 58.4% Female, Other states: 59.1% Female |
|  | Race / Ethnicity | CA: 6.3% Black, 51.4% Hispanic, 16.9% Other, 25.4% White  NJ: 14.7% Black, 21.1% Hispanic, 13.0% Other, 51.2% White  Other states: 15.7% Black, 17.7% Hispanic, 8.3% Other, 58.3% White |
|  | Income | CA: $73,852, NJ: $99,720, Other States: $68,368 |
|  | Insurance (e.g., Private, Medicaid, no insurance) | N/A |
|  | Education Status | CA: 31.4% High School or Less; 20.0% High School Grad; 26.4% Some College; 22.2% College Grad  NJ: 11.8% High School or Less; 24.7% High School Grad; 24.0% Some College; 39.4% College Grad  Other states: 16.2% High School or Less; 25.8% High School Grad; 29.9% Some College; 28.0% College Grad |
|  | Other Relevant Characteristics Tracked | Marital status, family size |
|  | Eligibility Criteria (e.g. inclusion / exclusion criteria) | Inclusion: Adults and children (<2 years) for whom the outcomes of interest were available  Exclusion: Adults who had never worked, and children with no working parent |
| **Intervention:** Paid Parental / Family Leave | Type of Leave | California Paid Family Leave (CA-PFL) and New Jersey’s PFL |
|  | Duration of Leave | 6 weeks for both CA and NJ |
|  | Pay While on Leave | CA: 55% wage replacement, up to $1,216 per week in 2018  NJ: 67% wage replacement, up to $637 per week in 2018 |
|  | Comparison Groups | CA and NJ vs. all non-PFL states (except RI) |
| **Outcome:**  Parent and Infant Health Outcomes | Health Indicator(s) measured | Parental mental health was measured using Kessler 6, a screening scale for psychological distress |
|  | Secondary Indicator(s) | N/A |
|  | Method of measurement (e.g. self-report, vital statistics, etc.) | Self-report |
| **Study Design** | Type of Quasi-Experimental Design | Difference-in-differences (DiD) |
|  | Additional details | N/A |
| **Data Source** | Type | National Health Interview Survey |
|  | Years of data source | 1997−2016 |
|  | Location(s) | CA, NJ and other states |
| **Methods** | Data Analysis | Difference-in-differences analysis |
|  | Covariates | Parent’s sex, race, educational attainment, marital status, family size, and age |
| **Main Findings** | Main Findings | - Exposure to PFL was associated with a 0.49-point decrease in psychological distress among parents (95% CI = −0.770, −0.21). - In stratified models, White, middle income, younger, and unmarried parents experienced improvements in psychological distress. - Black and Hispanic parents experienced increased psychological distress relative to White parents - Middle-income parents experienced greater improvements in psychological distress relative to low-income parents |
|  | Other findings (e.g. cost, implementation, feasibility, dissemination) | N/A |
|  | Implications of Research on Policy or Practice | Despite previous evidence that CA-PFL policy  narrows the gap in leave taking between economically  disadvantaged and advantaged parents, this study  found that White and middle-income parents  benefited more from the policy in terms of mental  health. |
| **Conclusions** |  | This study was among the first to use quasi-experimental  methods to examine the association of CA-PFL  policies with parental mental health, finding  positive impacts among parents |
| **Future Directions** |  | - Future work should examine the effects of recently implemented state policies that provide job protection and higher wage replacement for low-income parents to determine whether these resulted in greater leave taking and improved health among disadvantaged parents. - Future work should also consider whether variation in the implementation of PFL policies have differential impacts on vulnerable families. |
| **Strengths / Limitations** | Strengths of methods | N/A |
|  | Limitation of methods | Although parents who had never worked were excluded, the authors could not identify which parents were working at the time of their child’s birth or those who took leave after their child’s birth given the cross-sectional nature of the NHIS; this limitation means that the results of this study may underestimate the policy effects. |

**Kim (2024). “The Effects Of Paid Family Leave—Does It Help Fathers’ Health, Too?”**

| **Citation Details** | Author(s) | Jiyoon Kim |
| --- | --- | --- |
|  | Name of Article | “The Effects Of Paid Family Leave—Does It Help Fathers’ Health, Too?” |
|  | Author Affiliation | Department of Economics, Bryn Mawr College |
|  | Journal Name | *Journal of Population Economics* |
|  | Date of Publication | 2024 |
|  | Funding Source | N/A |
| **Study Aims** |  | To estimate the effects of California’s Paid Family Leave program on the health outcomes of both birthing parents and non-birthing partners |
| **Population** | Total sample size (n) | N/A |
|  | Age | Mothers  CA: Pre-PFL: 29.8 years, Post-PFL: 30.8 years; Non-PFL States: Pre-PFL 28.7 years, Post-PFL: 29.5 years  Fathers  CA: Pre-PFL: 31.9 years, Post-PFL: 32.2 years; Non-PFL States: Pre-PFL: 31.2 years, Post-PFL: 31.8 years |
|  | Gender of participants | N/A |
|  | Race / Ethnicity | Mothers  CA: Pre-PFL: 0.84 white, 0.03 Black, Post-PFL: 0.75 white, 0.09 Black, Non-PFL States: Pre-PFL 0.84 white, 0.03 Black, Post-PFL: 0.83 white, 0.13 Black  Fathers  CA: Pre-PFL: 0.84 white, 0.03 Black, Post-PFL: 0.90 white, 0.03 Black; Non-PFL States: Pre-PFL: 0.88 white, 0.08 Black; Post-PFL: 0.87 white, 0.07 Black |
|  | Income | Mothers  CA: Pre-PFL: 6383, Post-PFL: 5789; Non-PFL States: Pre-PFL 5210, Post-PFL: 5726  Fathers  CA: Pre-PFL: 5937 Post-PFL: 5629 ; Non-PFL States: 5282 Pre-PFL, Post-PFL: 5580 |
|  | Insurance (e.g., Private, Medicaid, no insurance) | N/A |
|  | Education Status | Mothers  CA: Pre-PFL: 0.15 Less than High School, 0.20 High School Grad, 0.32 Some College, Post-PFL: 0.10 Less than High School, 0.23 High School Grad, Some 0.28 College; Non-PFL States: Pre-PFL: 0.07 Less than High School, 0.27 High School Grad, 0.32 Some College, Post-PFL: 0.05 Less than High School, 0.16 High School Grad, 0.31 Some College  Fathers  CA: Pre-PFL: 0.24 Less than High School, 0.18 High School Grad, 0.29 Some College, Post-PFL: 0.23 Less than High School, 0.26 High School Grad, 0.25 Some College; Non-PFL States: Pre-PFL: 0.08 Less than High School, 0.29 High School Grad, 0.29 Some College; Post-PFL: 0.09 Less than High School, 0.25 High School Grad, 0.29 Some College |
|  | Other Relevant Characteristics Tracked | Marital Status |
|  | Eligibility Criteria (e.g. inclusion / exclusion criteria) | Inclusion: Parents with health topical modules that were collected anytime between nine months prior to childbirth and 20 months following childbirth. Mothers of reproductive age (18-45 years old) and fathers of all ages. Mothers or fathers who reported working at any point during the nine months prior to birth and had non-zero average earnings.  Exclusion: Self-employed parents, births in NJ |
| **Intervention:** Paid Parental / Family Leave | Type of Leave | California Paid Family Leave (CA-PFL) |
|  | Duration of Leave | 6 weeks |
|  | Pay While on Leave | 55% wage replacement |
|  | Comparison Groups | CA-PFL vs. other large states (Control 1 = FL, NY, PA, TX), Control 1 + neighboring states (Control 2 = Control 1 + AZ, NV, OR, WA), all non‐CA states plus D.C. (Control 3) |
| **Outcome:**  Parent and Infant Health Outcomes | Health Indicator(s) Measured | Self-reported health status, number of nights spent in the hospital, any sick days, and daily prescription drug usage for parents |
|  | Secondary Indicator(s) | N/A |
|  | Method of Measurement (e.g. self-report, vital statistics, etc.) | Self-report |
| **Study Design** | Type of Quasi-Experimental Design | Difference-in-differences |
|  | Additional details | N/A |
| **Data Source** | Data Source | Survey of Income and Program |
|  | Study Period | 1996 – 2013 |
|  | Location(s) | California and non-PFL states |
| **Methods** | Data Analysis | Difference-in-differences analysis |
|  | Covariates | Demographic and socioeconomic characteristics (e.g., age, marital status, employment, household characteristics) |
| **Main Findings** | Main Findings | - Birthing people showed a reduced likelihood of reporting any days sick (-6.51, p=0.000), fewer nights in the hospital (-0.058, p=0.000) and a lower likelihood of prescription drug use (-0.389, p=0.000). - Birthing people with access to CA-PFL spent 0.6 fewer nights in the hospital compared to those not exposed to PFL. - For non-birthing partners, CA-PFL led to an increase in “excellent” health by eight to 15 pp (0.149, p=0.000). - Non-birthing partners showed a reduced likelihood of reporting any sick days (-0.128, p=0.000) but no change in the number of days spent in the hospital (0.455, p=0.064) or the rate of prescription drug use (0.008, p=0.699). |
|  | Other Findings (e.g. cost, implementation, feasibility, dissemination) | N/A |
|  | Implications of Research on Policy or Practice | - PFL policies may improve health for both mothers and fathers, supporting expansion of leave policies to include non-birthing parents. - This study highlighted the role of PFL as a family-level health intervention, not just a maternal or infant policy. |
| **Conclusions** |  | - CA-PFL program improves health outcomes for both birthing parents and non-birthing partners, with particularly strong effects on self-reported health among non-birthing partners. |
| **Future Directions** |  | - Examination into the mechanisms through which PFL impacts birthing partner and non-birthing partner health outcomes would be beneficial in future studies. - Evaluating effects in states with newly implemented PFL programs beyond CA would enhance the generalizability of findings. |
| **Strengths / Limitations** | Strengths of Methods | - The quasi-experimental design strengthens causal inference. - Multiple comparison groups demonstrate that results are robust to multiple model specifications. - The use of nationally representative survey data increases the representation of the findings. |
|  | Limitation of Methods | - The use of self-reported health may introduce reporter bias. - As the data source does not report PFL use, the treatment in this study is exposure to PFL, therefore the results of this study may underestimate the true impact of the policy. - There are limited clinical health measures in this study. |

**Klevens et al (2017). “Paid Family Leave's Effect On Hospital Admissions For Pediatric Abusive Head Trauma.”**

| **Citation Details** | Author(s) | Joanne Klevens, MD, PhD, Feijun Luo, PhD, Likang Xu, MD, MS, Cora Peterson, PhD, and Natasha E. Latzman, PhD |
| --- | --- | --- |
|  | Name of Article | “Paid Family Leave's Effect On Hospital Admissions For Pediatric Abusive Head Trauma.” |
|  | Author Affiliation | National Center for Injury Prevention and Control, Centers for Disease Control and Prevention (CDC) |
|  | Journal Name | *Injury Prevention* |
|  | Date of Publication | 2017 |
|  | Funding Source | N/A |
| **Study Aims** |  | To evaluate whether California’s Paid Family Leave policy reduced hospital admissions for pediatric abusive head trauma (AHT) among infants < 1 year old. |
| **Population** | Total sample size (n) | N/A |
|  | Age | N/A |
|  | Gender of participants | N/A |
|  | Race / Ethnicity | N/A |
|  | Income | N/A |
|  | Insurance (e.g., Private, Medicaid, no insurance) | N/A |
|  | Education Status | N/A |
|  | Other Relevant Characteristics Tracked | N/A |
|  | Eligibility Criteria (e.g. inclusion / exclusion criteria) | Inclusion: Admissions for infants <1 year with a combination of International Classification of Diseases, Ninth Revision, Clinical Modification and External Cause of Injury diagnosis codes  Exclusion: Admissions in NJ, NY, OR, WA and SC |
| **Intervention:** Paid Parental / Family Leave | Type of Leave | California Paid Family Leave (CA-PFL) |
|  | Duration of Leave | 6 weeks |
|  | Pay While on Leave | 55% wage replacement |
|  | Comparison Groups | CA-PFL vs. seven non-PFL states (AZ, CO, FL, IA, MD, MA, WI) |
| **Outcome:** Parent and Infant Health Outcomes | Health Indicator(s) measured | Abusive Head Trauma (AHT) admissions among infants < 1 year old per 100,000 |
|  | Secondary Indicator(s) | N/A |
|  | Method of measurement (e.g. self-report, vital statistics, etc.) | Administrative data |
| **Study Design** | Type of Quasi-Experimental Design | Difference-in-difference |
|  | Additional details | N/A |
| **Data Source** | Data Source | Statewide Inpatient Databases from the Healthcare Cost and Utilization Project |
|  | Study Period | 1995 – 2011 |
|  | Location(s) | CA and non-PFL states |
| **Methods** | Data Analysis | Difference-in-difference analysis |
|  | Covariates | Unemployment rate and percentage of adults with education less than high school |
| **Main Findings** | Main Findings | - CA-PFL policy was significantly associated with a level decrease of 5.11 (p=0.035) in the AHT admissions per 100,000 infant < 1 year old. - While comparison states experienced increases in AHT rates during the years known as the Great Recession, CA did not. |
|  | Other Findings (e.g. cost, implementation, feasibility, dissemination) | - California did not experience the same increase in AHT admissions seen in comparison states during the Great Recession, suggesting PFL may buffer against economic stress-related child abuse risk |
|  | Implications of Research on Policy or Practice | - PFL may serve as a preventive measure against child maltreatment, particularly during periods of economic stress. - The findings of this study support the integration of paid leave into child abuse prevention and family support policies. |
| **Conclusions** |  | - CA-PFL was associated with reductions in AHT hospitalizations among infants, suggesting potential protective effects against severe child abuse. |
| **Future Directions** |  | - An examination into the mechanisms that link PFL to reductions in AHT hospital admissions for infants (e.g., reductions in parental stress) would provide further context for implementation in states considering PFL adoption. - Assessing the impact of PFL on AHT admissions for infants and children in other states would enhance the generalizability of the findings. |
| **Strengths / Limitations** | Strengths of Methods | - The use of longitudinal multi-state comparison groups strengthens the findings of this study. - The quasi-experimental design strengthens this study’s causal inference. |
|  | Limitation of Methods | - The relatively rare outcome of interest in this study reduces the statistical power of these findings. - The study also lacks individual characteristics as covariates. |

**Lee et al (2020). “The Effect Of California's Paid Family Leave Policy On Parent Health: A Quasi-Experimental Study.”**

| **Citation Details** | Author(s) | Bethany C. Lee,^a^ Sepideh Modrek,^b^ Justin S. White,^c,d,^ Akansha Batra,^d^ Daniel F. Collin,^e^ Rita Hamad,^c,e^ |
| --- | --- | --- |
|  | Name of Article | “The Effect Of California's Paid Family Leave Policy On Parent Health: A Quasi-Experimental Study.” |
|  | Author Affiliation | ^a^ University of Pennsylvania Law School  ^b^ Health Equity Institute, San Francisco State University  ^c^ Philip R. Lee Institute for Health Policy Studies, University of California San Francisco  ^d^ Department of Epidemiology & Biostatistics, University of California San Francisco  ^e^ Department of Family & Community Medicine, University of California San Francisco |
|  | Journal Name | *Social Science & Medicine* |
|  | Date of Publication | 2020 |
|  | Funding Source | National Institutes of Health (grant K08HL132106)  UCSF Hellman Fellows Fund  UCSF Irene Perstein Award  UCSF National Center of Excellence in Women's Health UCSF California Preterm Birth Initiative |
| **Study Aims** |  | To determine whether California’s paid family leave policy improves parent health outcomes |
| **Population** | Total sample size (n) | n = 6,690 families (631 CA, 6,059 other states) |
|  | Age | N/A |
|  | Gender of participants | CA: 52% Female, 48% Male  Other States: 53.1% Female, 26.9% Male  Overall: 53.0% Female, 27.0% Male |
|  | Race / Ethnicity | CA: 17.5% Black, 42.6% Hispanic 33.5% White, 6.3% Other  Other States: 31.2% Black, 6.6% Hispanic, 60.0% % White, 2.2% Other  Overall: 29.9% Black, 10.0% Hispanic, 57.5% White, 2.6% Other |
|  | Income | CA: $73,164; Other States: $67,341; Overall: 67,884 |
|  | Insurance (e.g., Private, Medicaid, no insurance) | N/A |
|  | Education Status | Overall: 10.9% High School or Less, 35.4% High School Grad, 28.9% Some College, 24.9% College Grad |
|  | Other Relevant Characteristics Tracked | N/A |
|  | Eligibility Criteria (e.g. inclusion / exclusion criteria) | Inclusion: Parents of children with a recorded state of birth, children under two years with at least one outcome of interest available  Exclusion: Households in which neither parent was employed one year before the child's birth, parents of multiple children under age two if one child was born under PFL policy and another was not |
| **Intervention:** Paid Parental / Family Leave | Type of Leave | California Paid Family Leave (CA-PFL) |
|  | Duration of Leave | 6 weeks |
|  | Pay While on Leave | 55% wage replacement |
|  | Comparison Groups | Non-PFL states |
| **Outcome:**  Parent and Infant Health Outcomes | Health Indicator(s) measured | Self-rated health, psychological distress, overweight and obesity, and alcohol use   - Parents' self-rated health was dichotomized as very good or excellent versus good, fair, or poor). - Parental mental health was assessed using the Kessler-6 (K6, range 0–24), a screening instrument for nonspecific psychological distress with higher scores indicating greater risk of mental illness (Prochaska et al., 2012). - They calculated parental body mass index (BMI) from self-reported height and weight. They analyzed BMI as binary variables for overweight (> 25 kg/m2) and obese (> 30 kg/m2) - They also examined whether participants drank any alcohol (binary) and whether they consumed three or more drinks daily (binary, zero for non-drinkers). |
|  | Secondary Indicator(s) | N/A |
|  | Method of measurement (e.g. self-report, vital statistics, etc.) | Self-report and state-level statistics |
| **Study Design** | Type of Quasi-Experimental Design | Difference-in-differences |
|  | Additional details | N/A |
| **Data Source** | Data Source | Panel Study of Income Dynamics |
|  | Study Period | 1993–2017 |
|  | Location(s) | California and other states |
| **Methods** | Data Analysis | - Difference-in-difference analysis |
|  | Covariates | - Individual characteristics: Parent gender, age, and educational attainment, household size, inflation-adjusted household income in the year before the child's birth, and the head of household's race - Fixed Effects, - State-level covariates: gross domestic product, unemployment rates, the percent of the adult population with less than a high school education, Aid to Families with Dependent Children/Temporary Assistant for Needy Families benefit size, Supplemental Nutritional Assistant Program benefit size, poverty rate, whether the governor was a Democrat, the state earned income tax credit rate, and the minimum wage |
| **Main Findings** | Main Findings | - Birthing people and non-birthing partners saw a 11 pp (0.11, 95% CI 5.2, 17.0) increased likelihood of being in “very good” or “excellent” self-rated health associated with CA-PFL. - Reductions in psychological distress (-0.79, 95% CI -1.26, -0.32), the likelihood of being overweight (-0.082, 95% CI -0.150, -0.016), any alcohol consumption and daily consumption of three or more drinks (-0.120, 95% CI -0.160, -0.070) were also found for both birthing people and non-birthing partners. - The improvements in health status and psychological distress were greater for birthing people, and the reduction in alcohol use was greater for non-birthing partners. |
|  | Other findings (e.g. cost, implementation, feasibility, dissemination) | N/A |
|  | Implications of Research on Policy or Practice | These findings suggest that California's PFL policy had positive impacts on several health outcomes, providing timely evidence to inform ongoing policy discussions at the federal and state levels. |
| **Conclusions** |  | This study provided some of the first estimates of the effects of CA-PFL on parent health, using longitudinal national data and a quasi-experimental study design |
| **Future Directions** |  | - Future studies should continue to examine treatment effect heterogeneity and assess whether PFL policies should be more equitably designed. - Future studies should examine the effects of more recently implemented state PFL policies to determine whether variation in policy implementation and generosity affects outcomes. - Future work could examine whether PFL policies have effects on long-term outcomes such as cardiovascular disease and premature death. |
| **Strengths / Limitations** | Strengths of Methods | The study’s quasi-experimental design enhanced the causal inference of PFL impacts. |
|  | Limitation of Methods | - The data source does not report leave use, therefore, PFL access is the treatment, which may underestimate the true impacts of the policy. - Several outcomes and covariates were self-reported, which may result in bias if these were associated with PFL access (treatment). - They were unable to examine effect heterogeneity based on parent characteristics such as race or ethnicity. |

**Montoya-Williams et al (2020). “The Impact Of Paid Family Leave In The United States On Birth Outcomes And Mortality In The First Year Of Life.”**

| **Citation Details** | Author(s) | Diana Montoya-Williams, Molly Passarella, and Scott A. Lorch |
| --- | --- | --- |
|  | Name of Article | “The Impact Of Paid Family Leave In The United States On Birth Outcomes And Mortality In The First Year Of Life.” |
|  | Author Affiliation | Division of Neonatology, Children's Hospital of Philadelphia |
|  | Journal Name | *Health Services Research* |
|  | Date of Publication | 2020 |
|  | Funding Source | National Institutes of Health, Grant R01 HD084819 and R01 HS018661 |
| **Study Aims** |  | To evaluate the effect of Paid Family Leave in California on statewide rates of preterm birth, low birthweight, postneonatal mortality, and overall infant mortality |
| **Population** | Total sample size (n) | n = 6,164,203 |
|  | Age | Pre-PFL: [Exposed]: 27.9 years; [Unexposed]: 27.5 years  Post-PFL: [Exposed]: 31.0 years; [Unexposed]: 30.0 years |
|  | Race / Ethnicity | Pre-PFL: [Exposed]: 12.3% Asian / Pacific Islander, 5.5% Black non-Hispanic, 45.8% Hispanic, 1.2% Other, 35.1% White non-Hispanic; [Unexposed]: 1.6% Asian / Pacific Islander, 12.5% Black non-Hispanic, 3.8% Hispanic, 6.7% Other, 75.5% White non-Hispanic  Post-PFL: [Exposed]: 13.6% Asian / Pacific Islander, 5.2% Black non-Hispanic, 49.1% Hispanic, 1.2% Other, 30.9% White non-Hispanic; [Unexposed]: 2.0% Asian / Pacific Islander, 14.4% Black non-Hispanic, 5.0% Hispanic, 4.4% Other, 74.2% White non-Hispanic |
|  | Income | N/A |
|  | Insurance (e.g., Private, Medicaid, no insurance) | Pre-PFL: [Exposed]: 53.5% Private, 43.0% Public, 0.0% Uninsured, 3.5% Other; [Unexposed]: 64.0% Private, 31.9% Public, 1.6% Uninsured, 2.4% Other  Post-PFL: [Exposed]: 48.9% Private, 47.9% Public, 0.0% Uninsured, 3.2% Other; [Unexposed]: 56.5% Private, 40.8% Public, 1.6% Uninsured, 1.1% Other |
|  | Education Status | Pre-PFL: [Exposed]: 11.9% No High School, 17.6% Some High School, 28.4% High School Grad/GED, 42.1% Some College; [Unexposed]: 1.9% No High School, 13.0% Some High School, 33.0% High School Grad/GED, 52.1% Some College  Post-PFL: [Exposed]: 10.3% No High School, 20.3% Some High School, 24.9% High School Grad/GED, 44.5% Some College; [Unexposed]: 2.1% No High School, 13.2% Some High School, 29.1% High School Grad/GED, 55.7% Some College |
|  | Other Relevant Characteristics Tracked | Gestational Age, Birthweight, Cesarean Section |
|  | Eligibility Criteria (e.g. inclusion / exclusion criteria) | Inclusion: Parents-infant dyads of singleton births  Exclusion: Infant gestational age was <23 weeks or >44 weeks, if birthweight was an outlier |
| **Intervention:** Paid Parental / Family Leave | Type of Leave | California Paid Family Leave (PFL) |
|  | Duration of Leave | 6 weeks |
|  | Pay While on Leave | 55% wage replacement |
|  | Comparison Groups | CA-PFL vs. two unexposed states (MO and PA) |
| **Outcome:**  Parent and Infant Health Outcomes | Health Indicator(s) measured | Infant health outcomes: preterm birth (live birth occurring prior to 37 weeks), low birthweight (infant weighing < 2500 g), postneonatal mortality (death occurring between 29 and 365 days of life), and overall infant mortality (death occurring at any point in the first year of life) |
|  | Secondary Indicator(s) | N/A |
|  | Method of Measurement (e.g. self-report, vital statistics, etc.) | Vital statistics |
| **Study Design** | Type of Quasi-Experimental Design | Difference-in-difference |
|  | Additional details | N/A |
| **Data Source** | Data Source | Live birth and death certificates from all in-hospital deliveries from state vital statistics |
|  | Study Period | 1999 – 2008 |
|  | Location(s) | CA-PFL vs. two unexposed states (MO and PA) |
| **Methods** | Data Analysis | Difference-in-difference analysis |
|  | Covariates | Time and state fixed effects, maternal age, education, insurance and race, median household income in home census tract, county unemployment rate, maternal comorbid conditions, perinatal complications, and congenital anomalies |
| **Main Findings** | Main Findings | - Compared to unexposed states, CA-PFL was shown to have decreased post-neonatal death by 12 percent (p=0.000). - There were no significant effects on the other health outcomes including pre-term birth, low birthweight, and infant mortality. - There were no differences in the effect by race/ethnicity or insurance status except for an increase in low birthweight among privately insured women in CA. - Reducing post-neonatal death by 1.94 per 10,000 live births was estimated to save 747 infant lives because of CA-PFL. |
|  | Other Findings (e.g. cost, implementation, feasibility, dissemination) | A parent who can have financial security after welcoming an infant may be able to invest more time in breastfeeding, attending preventive well-child visits, supervising children, or be less stressed overall, which may lead to lower rates of infant mortality. |
|  | Implications of Research on Policy or Practice | As other states have continued to enact their  own PFL policies that differ from CA-PFL in eligibility, maximum benefit, and duration, the estimated effect in this study may not be generalizable to all states. |
| **Conclusions** |  | Implementation of CA-PFL was associated with a 12 percent reduction in postneonatal mortality after adjusting for maternal and neonatal factors. |
| **Future Directions** |  | - Future studies should include similar evaluations of subsequently enacted statewide family leave policies since 2004. - PFL associations with improved breastfeeding and decreased abusive head trauma have been documented, but the rest of these hypothesized pathways for improved postneonatal mortality rates as a result of PFL must be further investigated. |
| **Strengths / Limitations** | Strengths of methods | - The quasi-experimental study design reduced bias and assisted in determining causality. |
|  | Limitation of methods | - Administrative and vital statistics data have both advantages and disadvantages. While such datasets allow for the analysis of large cohorts, there is a potential measurement error due to inaccurate measures in birth certificate data. - In addition, data restrictions did not allow for the inclusion of two important covariates in all three states: marital status and adequacy of prenatal care |

Morrissey et al (2024). “The Impacts of New York State's Paid Family Leave Policy on Parents' Sleep and Exercise.”

| **Citation Details** | Author(s) | Taryn W. Morrissey, Neko Michelle Castleberry, Aparna Soni |
| --- | --- | --- |
|  | Name of Article | “The Impacts of New York State's Paid Family Leave Policy on Parents' Sleep and Exercise.” |
|  | Author Affiliation | Department of Public Administration and Policy, School of Public Affairs, American University |
|  | Journal Name | *Maternal and Child Health Journal* |
|  | Date of Publication | 2024 |
|  | Funding Source | N/A |
| **Study Aims** |  | To assess changes in young parents’ health behaviors following implementation of New York State’s Paid Family Leave Program. |
| **Population** | Total sample size (n) | n = 136,525 |
|  | Age | NY: Pre-PFL: 26 ; Post-PFL: 26  Comparison States: Pre-PFL: 26 ; Post-PFL: 26 |
|  | Gender of participants | NY: Pre-PFL: 58.8% Female, 41.2% Male; Post-PFL: 61.1% Female, 38.9% Male  Comparison States: Pre-PFL: 58.9% Female, 40.3% Male; Post-PFL: 60.8% Female, 39.2% Male |
|  | Race / Ethnicity | NY: Pre-PFL: 60.0% White, 24.1% Black, 15.9% Other, 27.8% Hispanic; Post-PFL: 55.5% White, 22.4% Black, 21.8% Other, 29.4% Hispanic  Comparison States: Pre-PFL: 68.6% White, 15.0% Black, 16.4% Other, 22.7% Hispanic; Post-PFL: 67.4% White, 15.8% Black, 16.8% Other, 24.7% Hispanic |
|  | Income | NY: Pre-PFL: 57.9% Less than $35K, 13.6% $35K - $50K, 28.5% $50K+; Post-PFL: 50.4% Less than $35K, 12.3% $35K - $50K, 37.3% $50K+  Comparison States: Pre-PFL: 50.5% Less than $35K, 15.0% $35K - $50K, 34.6% $50K+; Post-PFL: 42.7% Less than $35K, 14.2% $35K - $50K, 43.1% $50K+ |
|  | Insurance (e.g., Private, Medicaid, no insurance) | NY: Pre-PFL: 72.0%; Post-PFL: 76.2%  Comparison States: Pre-PFL: 79.4%; Post-PFL: 79.1% |
|  | Education Status | NY: Pre-PFL: 50.4% High School or Less, 49.1% Some College or More; Post-PFL: 50.2% High School or Less, 49.9% Some College or More  Comparison States: Pre-PFL: 46.3% High School or Less, 53.7% Some College or More; Post-PFL: 46.2% High School or Less, 53.8% Some College or More |
|  | Other Relevant Characteristics Tracked | Family Structure, Employment, Number of Children |
|  | Eligibility Criteria (e.g. inclusion / exclusion criteria) | Inclusion: Adults between the ages of 21 and 30 living in a household with at least one child under the age of 18 years living.  Exclusion: Adults and infants in CA, NJ or RI. |
| **Intervention:** Paid Parental / Family Leave | Type of Leave | New York States Paid Family Leave policy (NYSPFL) |
|  | Duration of Leave | 12 weeks |
|  | Pay While on Leave | 67% wage replacement |
|  | Comparison Groups | New York vs. synthetic control |
| **Outcome:**  Parent and Infant Health Outcomes | Health Indicator(s) measured | A self-reported binary measure of exercise in the past month and average hours of sleep per day (average 24-hour period) |
|  | Secondary Indicator(s) | N/A |
|  | Method of measurement (e.g. self-report, vital statistics, etc.) | Self-report |
| **Study Design** | Type of Quasi-Experimental Design | Difference-in-difference with synthetic control |
|  | Additional details | N/A |
| **Data Source** | Data Source | Behavioral Risk Factor Surveillance System |
|  | Study Period | 2011 – 2019 |
|  | Location(s) | NY |
| **Methods** | Data Analysis | Difference-in-difference analysis |
|  | Covariates | - State characteristics: Rates of unem­ployment, poverty, whether a state has an EITC, and the percent of state population for those: under 18 years, over 64 years, White, Black, Hispanic, and female. - Individual characteristics: Age, gender, race, eth­nicity, education, marital/cohabitation status, employment status, household income, number of children, and health insurance status, state of residence, and month-year of interview. |
| **Main Findings** | Main Findings | - NYSPFL increased the likelihood of exercise among birthing people, single parents (0.11, 95% CI 0.048, 0.255), and low-income parents (0.072, CI -0.052, 0.289). - Non-birthing partners, single parents, and those with two or more children showed an increase in daily sleep between 14 and 21 min per day. - There was no significant change in sleep for birthing people as a result of PFL. |
|  | Other Findings (e.g. cost, implementation, feasibility, dissemination) | N/A |
|  | Implications of Research on Policy or Practice | The findings of this study suggest that PFL policies may improve not only infant/parent bonding and labor market outcomes, but also parental health behaviors such as sleep and exercise. |
| **Conclusions** |  | Implementation of NYSPFL is associated with positive impacts on parental health behaviors, supporting broader public health benefits beyond physical health outcomes to also include well-being outcomes. |
| **Future Directions** |  | Assessing the long-term impact of NYSPFL would enhance the findings of this study.  An examination into the pathways through which PFL impacts parental behavioral health may contribute to stronger rationales to implement PFL in more states.  An understanding of leave generosity (e.g. leave length and wage replacement) may promote the extension of PFL in existing states with shorter PFL policies. |
| **Strengths / Limitations** | Strengths of Methods | The use of large-scale population data enhances the representation of this study.  The quasi-experimental design strengthens the causal inference. |
|  | Limitation of Methods | There is limited generalizability as this study focuses on one state of interest.  Reliance on self-reported outcomes may introduce reporter bias.  The data source does not report leave use, so the exposure of interest in this study is exposure to PFL. |

Pihl & Basso (2019). “Did California Paid Family Leave Impact Infant Health?”

| **Citation Details** | Author(s) | Ariel Marek Pihl^a^ and Gaetano Basso^b^ |
| --- | --- | --- |
|  | Name of Article | “Did California Paid Family Leave Impact Infant Health?” |
|  | Author Affiliation | ^a^ Department of Economics at the University of Gothenburg  ^b^ Economics, Statistics and Research, Labour Market and Structural Economic Analysis Division, Bank of Italy |
|  | Journal Name | *Journal of Policy Analysis and Management* |
|  | Date of Publication | 2019 |
|  | Funding Source | University of California Davis Department of Economics |
| **Study Aims** |  | To evaluate whether California’s Paid Family Leave program improved a range of infant health outcomes using administrative hospitalization data. |
| **Population** | Total sample size (n) | n = 1,101,681 |
|  | Age | CA: 29.2 years  AZ, NY and WA: 29.3 years |
|  | Gender of participants | N/A |
|  | Race / Ethnicity | CA: 0.40 White, 0.40 Hispanic  AZ, NY and WA: 0.58 White, 0.22 Hispanic |
|  | Income | CA: $60,102  AZ, NY and WA: $59,943 |
|  | Insurance (e.g., Private, Medicaid, no insurance) | N/A |
|  | Education Status | N/A |
|  | Other Relevant Characteristics Tracked | Working, Worked Last Year, Works 35+ Hours |
|  | Eligibility Criteria (e.g. inclusion / exclusion criteria) | Inclusion: Working parents in CA with a baby born after July 31, 2003, CA infants under one year of age  Exclusion: Infants who have been hospitalized since birth |
| **Intervention:** Paid Parental / Family Leave | Type of Leave | California Paid Family Leave (CA-PFL) |
|  | Duration of Leave | 6 weeks |
|  | Pay While on Leave | 55% wage replacement |
|  | Comparison Groups | CA vs. three non-PFL states (AZ, NY, WA) |
| **Outcome:**  Parent and Infant Health Outcomes | Health Indicator(s) measured | Infant hospital admissions for lower respiratory, upper respiratory, skin infections, gastrointestinal infections, and cancer |
|  | Secondary Indicator(s) | N/A |
|  | Method of measurement (e.g. self-report, vital statistics, etc.) | Administrative data |
| **Study Design** | Type of Quasi-Experimental Design | Difference-in-difference |
|  | Additional details | N/A |
| **Data Source** | Data Source | California Office of Statewide Health Planning and Development |
|  | Study Period | 2000 – 2007 |
|  | Location(s) | CA and AZ, NY, WA |
| **Methods** | Data Analysis | Difference-in-difference analysis |
|  | Covariates | Unemployment rate, average precipitation, average minimum and maximum temperature, and average fine particulate matter concentration |
| **Main Findings** | Main Findings | - The introduction of CA-PFL showed a decline in all hospitalization among infants by six percent (-0.062, p=0.017). - Specifically, infant hospital admissions for upper respiratory complications declined by 25 percent (-0.247, p=0.078) and by 9 percent (-0.088, p=0.034) for gastrointestinal infections. - There was no statistically significant change in infant hospital admissions for lower respiratory complications or cancer. - A six percent decrease in hospitalizations translated to 4,380 fewer hospitalizations. |
|  | Other Findings (e.g. cost, implementation, feasibility, dissemination) | N/A |
|  | Implications of Research on Policy or Practice | The findings of this study suggest that PFL improves infant health through improved parental caregiving, which may ultimately and potentially reduce healthcare utilization. |
| **Conclusions** |  | CA-PFL is associated with improvements in infant health, as evidenced by reductions in hospital admissions for conditions sensitive to parental care. |
| **Future Directions** |  | - An examination into long-term developmental outcomes could be beneficial based on the findings of this study. - A cost-benefit analysis of reduced hospitalizations is also a worthwhile investigation. |
| **Strengths / Limitations** | Strengths of Methods | - The use of a large-scale administrative data set enhances the statistical power of this study’s findings. - The quasi-experimental design strengthens the causal inference of PFL on infant hospital admissions. |
|  | Limitation of Methods | - There is limited investigation into the mechanisms through which PFL improves several indicators related to infant hospitalizations. - Hospital admissions are also extreme events, and this study does not capture harmful but not as extreme medical events to infants. - The generalizability of this study is limited due to the geographic focus on CA. |
