# Supplementary material for: The impact of paid family leave on parent and infant health outcomes: a systematic review of quasi-experimental research
Source: BMC Public Health. 2026 Jul 14;26:2914. doi: 10.1186/s12889-026-27709-2 (PMC13651160; doi:10.1186/s12889-026-27709-2)
Supplement: Supplementary file 5 [file 12889_2026_27709_MOESM5_ESM.docx]

PRISMA Diagram

Studies from databases/registers **(n = 5543)**

Scopus (n = 2376)

PsycINFO (n = 1434)

PubMed (n = 939)

CINAHL (n = 794)

References from other sources **(n = 0)**

**Identification**

Studies included in review **(n = 10)**

Studies excluded following title and abstract screening **(n = 3056)**

Studies not retrieved **(n = 0)**

Studies assessed for eligibility **(n = 31)**

Studies sought for retrieval **(n = 31)**

Studies screened **(n = 3087)**

Studies excluded **(n = 21)**

Not peer-reviewed (n = 1)

Not based in the U.S. (n = 1)

Not health outcomes (n = 7)

Not quasi-experimental design (n = 10)

Not paid family leave following childbirth (n = 1)

The population of focus is not parents or infants

(n = 1)

References removed **(n = 2456)**

Duplicates identified manually (n = 21)

Duplicates identified by Covidence (n = 2435)

Marked as ineligible by automation tools (n = 0)

Other reasons (n = 0)

**Screening**

**Included**
