# Supplementary material for: The impact of paid family leave on parent and infant health outcomes: a systematic review of quasi-experimental research
Source: BMC Public Health. 2026 Jul 14;26:2914. doi: 10.1186/s12889-026-27709-2 (PMC13651160; doi:10.1186/s12889-026-27709-2)
Supplement: Supplementary file 6 [file 12889_2026_27709_MOESM6_ESM.docx]

ROBINS-I Assessment for Parent Physical Health

| **Study** | **Confounding** | **Classification of policy** | **Selection of participants** | **Missing data** | **Outcome Measurement** | **Selection of reported result** | **Overall** |
| --- | --- | --- | --- | --- | --- | --- | --- |
| **Kim 2024** | **Low** (except for concerns about uncontrolled confounding)  The authors control for all the important confounding factors, which were measured validly and reliably. | **Low** The authors use an appropriate analysis method to control for time-varying and baseline confounding (e.g. DD). As the data source reports PFL use, there is low risk for classification errors (e.g. Survey of Income and Program Participation). | **Low** Analysis begins at the start of the policy, and selection of participants was not based on post-policy participant characteristics. | **Serious** The study provides no information about the extent of missing data. | **Low** Measurement of the outcome did not differ between policy groups, and assessors were blinded to policy status. | **Low** Results were reported according to the analysis plan and there is only one way to interpret multiple outcome measurements, analyses and subgroups. | **Serious** Despite the majority of low risk of bias ratings across the domains, the study provides no information about the extent of missing data and warrants a serious risk of bias rating. |
| **Lee *et al.* 2021** | **Low** (except for concerns about uncontrolled confounding)  The authors control for all the important confounding factors, which were measured validly and reliably. | **Moderate** The authors use an appropriate analysis method to control for necessary confounding variables (e.g. DD). However, non-differential misclassification of intervention status is likely since the data source does not report participant PFL use (e.g. Panel Study of Income Dynamics) | **Low** Analysis begin at the start of the intervention strategies being compared and selection of participants was not based on participant characteristics observed after the start of intervention. | **Low**  Complete data on intervention status and outcome variables was available for nearly all participants but important confounding variables were missing (e.g. up to 25%). A complete case analysis was not conducted, and analysis was based on imputing missing values. It is reasonable to assume that variables were missing at random, and there was an appropriate imputation technique used. | **Low** Measurement of the outcome did not differ between intervention groups, and assessors were blinded to intervention status. | **Low** Results were reported according to the analysis plan and there is only one way to interpret multiple outcome measurements, analyses and subgroups. | **Moderate** Only one domain was at a moderate risk of bias with all remaining domains being low risk of bias. |
| **Morrissey et al. 2024** | **Low** (except for concerns about uncontrolled confounding)  The authors control for all the important confounding factors, which were measured validly and reliably. | **Moderate** The authors use an appropriate analysis method to control for necessary confounding variables (e.g. DD with Synthetic Control). However, non-differential misclassification of intervention status is likely since the data source does not report participant PFL use (e.g. Behavioral Risk Factor Surveillance System (BRFSS) | **Low** Analysis begin at the start of the intervention strategies being compared and selection of participants was not based on participant characteristics observed after the start of intervention. | **Serious** The study provides no information about the extent of missing data. | **Low** Measurement of the outcome did not differ between intervention groups, and assessors were blinded to intervention status. | **Low** Results were reported according to the analysis plan and there is only one way to interpret multiple outcome measurements, analyses and subgroups. | **Serious** Despite the majority of low risk of bias ratings across the domains, the study provides no information about the extent of missing data and warrants a serious risk of bias rating. |

ROBINS-I Assessment for Parent Mental Health

| **Study** | **Confounding** | **Classification of policy** | **Selection of participants** | **Missing data** | **Outcome Measurement** | **Selection of reported result** | **Overall** |
| --- | --- | --- | --- | --- | --- | --- | --- |
| **Bullinger 2019** | **Low** (except for concerns about uncontrolled confounding)  The authors control for all the important confounding factors, which were measured validly and reliably. | **Moderate** The authors use an analysis method that was appropriate to control for time-varying and baseline confounding (e.g. DD and DDD). However, non-differential misclassification of intervention status is somewhat likely since there is no way to confirm that PFL was used by participant (e.g. National Survey of Children’s Health and Behavioral Risk Factor Surveillance System). | **Low** Analysis begin at the start of the intervention strategies being compared and selection of participants was not based on participant characteristics observed after the start of intervention. | **Serious** The study provides no information about the extent of missing data. | **Low** Measurement of the outcome did not differ between intervention groups, and assessors were blinded to intervention status. | **Low** Results were reported according to the analysis plan and there is only one way to interpret multiple outcome measurements, analyses and subgroups. | **Serious** Despite the majority of low risk of bias ratings across the domains, the study provides no information about the extent of missing data and warrants a serious risk of bias rating. |
| **Doran *et al.* 2020** | **Low** (except for concerns about uncontrolled confounding)  The authors control for all the important confounding factors, which were measured validly and reliably. | **Moderate** The authors use an analysis method that was appropriate to control for time-varying and baseline confounding (e.g. DD with Synthetic Control). However, non-differential misclassification of intervention status is likely since the data source does not report PFL use by participants (e.g. National Health Interview Survey) | **Low** Analysis begin at the start of the intervention strategies being compared and selection of participants was not based on participant characteristics observed after the start of intervention. | **Low**  Complete data on intervention status, outcome and important confounding variables was available for nearly all participants (92% of all participants) | **Low** Measurement of the outcome did not differ between intervention groups, and assessors were blinded to intervention status. | **Low** Results were reported according to the analysis plan and there is only one way to interpret multiple outcome measurements, analyses and subgroups. | **Moderate** Only one domain was at a moderate risk of bias with all remaining domains being low risk of bias. |
| **Irish *et al.* 2021** | **Low** (except for concerns about uncontrolled confounding)  The authors control for all the important confounding factors, which were measured validly and reliably. | **Moderate** The authors use an appropriate analysis method to control for necessary confounding variables (e.g. DD). However, non-differential misclassification of intervention status is likely since the data source does not report PFL use by participants (e.g. National Health Interview Survey) | **Low** Analysis begin at the start of the intervention strategies being compared and selection of participants was not based on participant characteristics observed after the start of intervention. | **Low**  Complete data on intervention status, outcome and important confounding variables was available for nearly all participants (95% of all participants) | **Low** Measurement of the outcome did not differ between intervention groups, and assessors were blinded to intervention status. | **Low** Results were reported according to the analysis plan and there is only one way to interpret multiple outcome measurements, analyses and subgroups. | **Moderate** Only one domain was at a moderate risk of bias with all remaining domains being low risk of bias. |
| **Lee *et al.* 2021** | **Low** (except for concerns about uncontrolled confounding)  The authors control for all the important confounding factors, which were measured validly and reliably. | **Moderate** The authors use an appropriate analysis method to control for necessary confounding variables (e.g. DD). However, non-differential misclassification of intervention status is likely since the data source does not report participant PFL use (e.g. Panel Study of Income Dynamics) | **Low** Analysis begin at the start of the intervention strategies being compared and selection of participants was not based on participant characteristics observed after the start of intervention. | **Low**  Complete data on intervention status and outcome variables was available for nearly all participants but important confounding variables were missing (e.g. up to 25%). A complete case analysis was not conducted, so analysis was based on imputing missing values. It is reasonable to assume that variables were missing at random, and there was an appropriate imputation technique used. | **Low** Measurement of the outcome did not differ between intervention groups, and assessors were blinded to intervention status. | **Low** Results were reported according to the analysis plan and there is only one way to interpret multiple outcome measurements, analyses and subgroups. | **Moderate** Only one domain was at a moderate risk of bias with all remaining domains being low risk of bias. |

ROBINS-I Assessment for Infant Physical Health

| **Study** | | **Confounding** | | **Classification of policy** | | **Selection of participants** | | **Missing data** | | **Outcome Measurement** | | | **Selection of reported result** | **Overall** |
| --- | --- | --- | --- | --- | --- | --- | --- | --- | --- | --- | --- | --- | --- | --- |
| **Bullinger 2019** | | **Low** (except for concerns about uncontrolled confounding)  The authors control for all important confounding factors, which were measured validly and reliably. | | **Moderate** The authors use an analysis method that was appropriate to control for time-varying and baseline confounding (e.g. DD and DDD). However, non-differential misclassification of policy status is somewhat likely since there is no way to confirm that PFL was used by participants via the data source (ex. National Survey of Children’s Health). | | **Low** Analysis begin at the start of the policy, and selection of participants was not based on their characteristics observed after the implementation of the policy. | | **Serious** The study provides no information about the extent of missing data. | | **Low** Measurement of the outcome did not differ between policy groups, and assessors were blinded to policy status. | | | **Low** Results were reported according to the analysis plan and there is only one way to interpret multiple outcome measurements, analyses and subgroups. | **Serious** Despite the majority of low risk of bias rating across the domains, the study provides no information about the extent of missing data and warrants a serious risk of bias rating. |
| **Chen 2023** | | **Low** (except for concerns about uncontrolled confounding)  The authors control for all the important confounding factors, which were measured validly and reliably. | | **Moderate** An appropriate analysis method to control for time-varying and baseline confounding (e.g. DD) was employed. However, non-differential misclassification of policy status is likely since the data source does not report whether PFL was used by participants (e.g. National Vital Statistics Survey). | | **Low** Analysis begins at the start of the policy, and selection of participants was not based on their characteristics post-policy. | | **Serious** The study provides no information about the extent of missing data. | | **Low** Outcome measurements did not differ between policy groups, and assessors did not know policy status. | | | **Low** Results were reported according to the analysis plan and there is only one way to interpret multiple outcome measurements, analyses and subgroups. | **Serious** Despite the majority of low risk of bias rating across the domains, the study provides no information about the extent of missing data and warrants a serious risk of bias rating. |
| **Klevens *et al.* 2016** | | **Low** (except for concerns about uncontrolled confounding)  The authors control for all the important confounding factors, which were measured validly and reliably. | | **Moderate** The authors use an analysis method that was appropriate to control for time-varying and baseline confounding (e.g. DD). However, non-differential misclassification of policy status is somewhat likely since there is no way to confirm that PFL was used by participants (e.g. Statewide Inpatient Databases from the Healthcare Cost and Utilization Project). | | **Low** Analysis begin at the start of the policy, and selection of participants was not based on participant characteristics from after the start of policy. | | **Serious** The study provides no information about the extent of missing data. | | **Low** Measurement of the outcome did not differ between policy groups, and assessors were blinded to policy status. | | | **Low** Results were reported according to the analysis plan and there is only one way to interpret multiple outcome measurements, analyses and subgroups. | **Serious** Despite the majority of low risk of bias rating across the domains, the study provides no information about the extent of missing data and warrants a serious risk of bias rating. |
| **Montoya-Williams *et al.* 2020** | | **Low** (except for concerns about uncontrolled confounding)  The authors control for all the important confounding factors, which were measured validly and reliably. | | **Moderate** The authors use an appropriate analysis method to control for time-varying and baseline confounding (e.g. DD). However, non-differential misclassification of treatment status is somewhat likely since the data source does not gather if PFL was used by individual participants (e.g. birth and death certificate data from CA, MO, and PA). | | **Low** Analysis begins at the start of the policy, and selection of participants was not based on post-policy participant characteristics. | | **Serious** The study provides no information about the extent of missing data. | | **Low** Measurement of the outcome did not differ between policy groups, and assessors were blinded to policy status. | | | **Low** Results were reported according to the analysis plan and there is only one way to interpret multiple outcome measurements, analyses and subgroups. | **Serious** Despite the majority of low risk of bias rating across the domains, the study provides no information about the extent of missing data and warrants a serious risk of bias rating. |
| **Pihl & Basso 2018** | | **Low** (except for concerns about uncontrolled confounding)  The authors control for all the important confounding factors, which were measured validly and reliably. | | **Moderate** The authors use an appropriate analysis method to control for time-varying and baseline confounding (e.g. DD). However, non-differential misclassification of treatment status is likely since the data source does not report if PFL was used by individual participants (e.g. The Office of Statewide Health Planning and Development) | | **Low** Analysis begins at the start of the policy, and selection of participants was not based on post-policy participant characteristics. | | **Serious** The study provides no information about the extent of missing data. | | **Low** Measurement of the outcome did not differ between policy groups, and assessors were blinded to policy status. | | | **Low** Results were reported according to the analysis plan and there is only one way to interpret multiple outcome measurements, analyses and subgroups. | **Serious** Despite the majority of low risk of bias ratings across the domains, the study provides no information about the extent of missing data and warrants a serious risk of bias rating. |
|  |  | |  | |  | |  | |  | |  |  | | |
